# Osthole ameliorates wear particle-induced osteogenic impairment by mitigating endoplasmic reticulum stress via PERK signaling cascade

**DOI:** 10.1186/s10020-024-01034-z

**Published:** 2024-12-20

**Authors:** Xin Yu, Juan Jiang, Cheng Li, Yang Wang, Zhengrong Ren, Jianlun Hu, Tao Yuan, Yongjie Wu, Dongsheng Wang, Ziying Sun, Qi Wu, Bin Chen, Peng Fang, Hao Ding, Jia Meng, Hui Jiang, Jianning Zhao, Nirong Bao

**Affiliations:** 1 Department of Orthopedics, Nanjing Jinling Hospital, Affiliated Hospital of Medical School, Nanjing University, Nanjing, China; 2 State Key Laboratory of Pharmaceutical Biotechnology, School of Life Sciences, Nanjing University, Nanjing, China; 3 State Key Laboratory of Pharmaceutical Biotechnology, Jiangsu Key Laboratory of Molecular Medicine, Medical School, Nanjing University, Nanjing, China; 4 Department of Spine Surgery, Center of Orthopedics, Daping Hospital, Army Medical University (Third Military Medical University), Chongqing, China; 5 Department of Vascular Surgery, Beijing Tsinghua Changgung Hospital, School of Clinical Medicine, Tsinghua University, Beijing, China; 6 Berlin Institute of Health at Charité - Universitätsmedizin Berlin, Julius Wolff Institute, Berlin, Germany; 7 Department of Orthopedics, Nanjing Hospital of Chinese Medicine, Nanjing University of Chinese Medicine, Nanjing, China

**Keywords:** Osthole, Aseptic loosening, Osteolysis, Osteoblast, ER stress, PERK signaling cascade

## Abstract

**Background:**

Periprosthetic osteolysis and subsequent aseptic loosening are the leading causes of failure following total joint arthroplasty. Osteogenic impairment induced by wear particles is regarded as a crucial contributing factor in the development of osteolysis, with endoplasmic reticulum (ER) stress identified as a key underlying mechanism. Therefore, identifying potential therapeutic targets and agents that can regulate ER stress adaption in osteoblasts is necessary for arresting aseptic loosening. Osthole (OST), a natural coumarin derivative, has demonstrated promising osteogenic properties and the ability to modulate ER stress adaption in various diseases. However, the impact of OST on ER stress-mediated osteogenic impairment caused by wear particles remains unclear.

**Methods:**

TiAl_6_V_4_ particles (TiPs) were sourced from the prosthesis of patients who underwent revision hip arthroplasty due to aseptic loosening. A mouse calvarial osteolysis model was utilized to explore the effects of OST on TiPs-induced osteogenic impairment in vivo. Primary mouse osteoblasts were employed to investigate the impact of OST on ER stress-mediated osteoblast apoptosis and osteogenic inhibition induced by TiPs in vitro. The mechanisms underlying OST-modulated alleviation of ER stress induced by TiPs were elucidated through Molecular docking, immunochemistry, PCR, and Western blot analysis.

**Results:**

In this study, we found that OST treatment effectively mitigated TiAl_6_V_4_ particles (TiPs)-induced osteolysis by enhancing osteogenesis in a mouse calvarial model. Furthermore, we observed that OST could attenuate ER stress-mediated apoptosis and osteogenic reduction in osteoblasts exposed to TiPs in vitro and in vivo. Mechanistically, we demonstrated that OST exerts bone-sparing effects on stressed osteoblasts upon TiPs exposure by specifically suppressing the ER stress-dependent PERK signaling cascade.

**Conclusion:**

Osthole ameliorates wear particle-induced osteogenic impairment by mitigating endoplasmic reticulum stress via PERK signaling cascade. These findings suggest that OST may serve as a potential therapeutic agent for combating wear particle-induced osteogenic impairment, offering a novel alternative strategy for managing aseptic prosthesis loosening.

**Supplementary Information:**

The online version contains supplementary material available at 10.1186/s10020-024-01034-z.

## Introduction

Aseptic prosthesis loosening is the predominant long-term complication following total joint arthroplasty, constituting the primary cause of implant failure and revision surgery (Carender et al. 2024). Periprosthetic osteolysis, initiated by wear particles generated from bearing surfaces during extended usage periods, is the principal etiological factor contributing to aseptic loosening (Anil et al. 2022). Normal bone metabolism requires a balance between osteoclast-driven bone resorption and osteoblast‐mediated bone formation. Osteoblasts play a pivotal role in the overall process of bone remodeling, and any alternations in their quantity and functionality directly influence the progression of osteolysis (O’Neill et al. 2013). Wear particles have been demonstrated to adversely affect osteoblast viability and function, resulting in reduced periprosthetic bone formation and impaired bone remodeling (Zhang et al. 2020). Therefore, maintaining the survival and functionality of osteoblasts emerges as a promising avenue for intervention in both the treatment and prevention of periprosthetic osteolysis and subsequent aseptic loosening.

The endoplasmic reticulum (ER) is an essential membranous organelle in eukaryotic cells, primarily responsible for protein synthesis, folding, and maturation (Schwarz and Blower 2016). Proper ER function is fundamental for maintaining protein homeostasis, which is crucial for cellular function and survival. Disturbances in cellular homeostasis can lead to the accumulation of unfolded or misfolded proteins within the ER lumen, resulting in a condition commonly known as ER stress (Wang and Kaufman 2016). In response, the unfolded protein response (UPR) is activated to mitigate ER stress and restore ER proteostasis (Hetz 2012). However, when the UPR fails to restore ER function, prolonged ER stress ultimately triggers cellular apoptosis (Hetz and Papa 2018). The UPR consists of a series of signaling and transcriptional events initiated by three ER transmembrane sensors: protein kinase R-like ER kinase (PERK), inositol-requiring enzyme-1α (IRE1α), and activating transcription factor 6 (ATF6). Among the components of UPR, C/EBP-homologous protein (CHOP), a downstream transcription factor that serves as a prominent link among all three branches of the UPR, has been regarded as the major culprit in ER stress-mediated apoptosis (Szegezdi et al. 2006; Tabas and Ron 2011). Given that CHOP is predominantly activated through the PERK branch of the UPR, the PERK-CHOP axis seems of importance for cellular survival during ER stress (Hetz 2012; Tabas and Ron 2011). Previous studies by our laboratory have demonstrated that ER stress mediates wear particle-induced osteogenic impairment and periprosthetic osteolysis (Wang et al. 2013; Yu et al. 2023a, b, 2024a, b). These findings suggest that targeting ER stress could serve as a promising therapeutic strategy for preventing and treating aseptic loosening. Specifically, interventions aimed at modulating the UPR and mitigating the detrimental effects of prolonged ER stress could potentially improve outcomes in conditions associated with wear particle generation and subsequent osteogenic impairment.

**Fig. 1:**
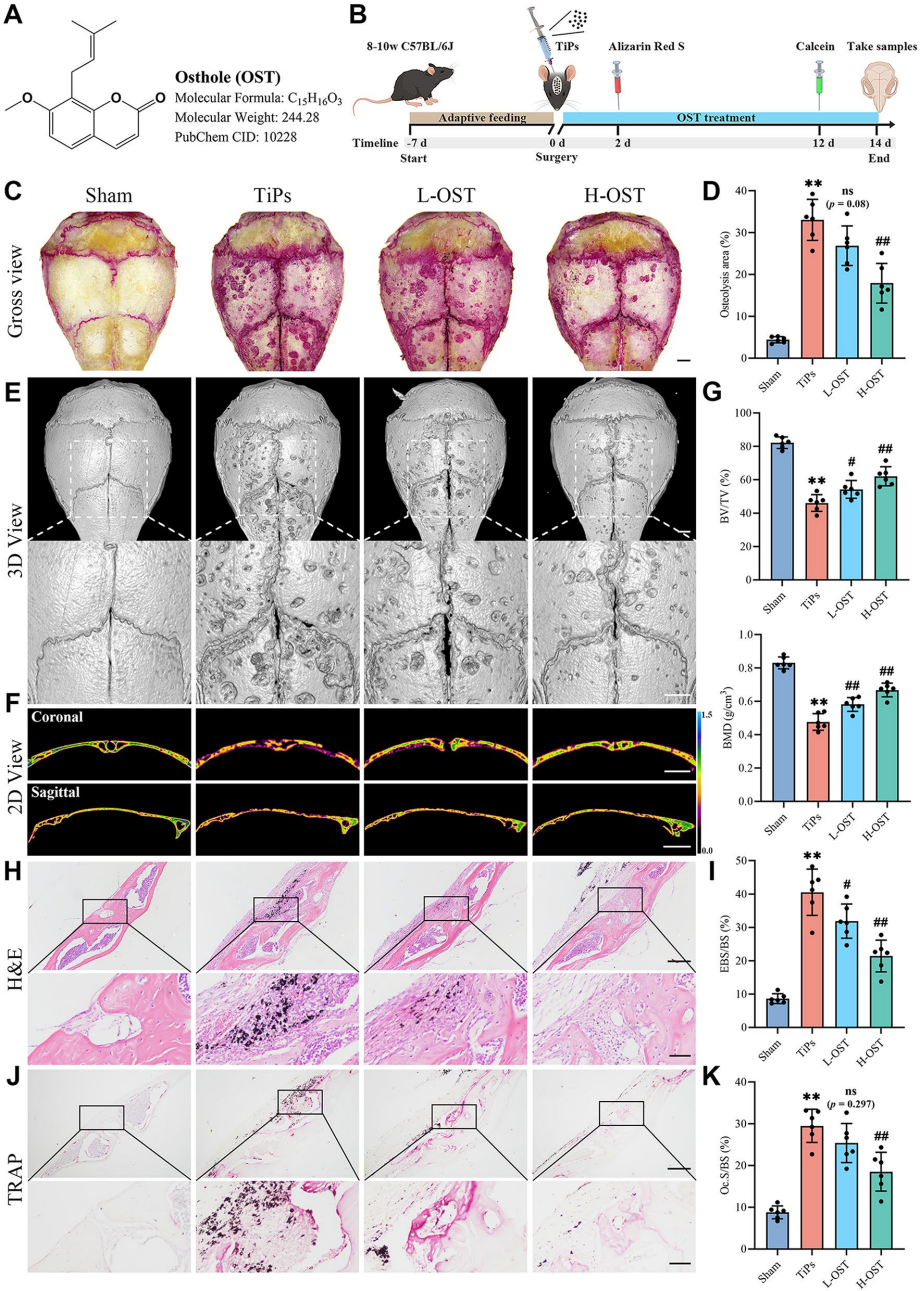
OST treatment ameliorated TiPs-induced osteolysis in a mouse calvarial model. (**A**) The chemical structure, molecular formula, molecular weight, and PubChem CID of Osthole (OST). (**B**) Timeline of in vivo experimental design. (**C**) Gross view of TRAP-stained whole calvaria. Scale bar, 1 mm. (**D**) Quantification of the percentage of osteolysis area in calvaria (%). (**E**) Representative micro-CT three-dimensional (3D) reconstructed images of mouse calvaria. Scale bar, 1 mm. (**F**) Representative pseudo-color images of micro-CT two-dimensional (2D) reconstruction of mouse calvaria in the coronal and sagittal planes. Scale bar, 1 mm (coronal), 2 mm (sagittal). The gradient color scale indicated tissue density (g/cm^3^). (**G**) Quantitative analysis of bone morphometric parameters, including BV/TV (%) and BMD (g/cm^3^). (**H**) Representative H&E staining images of calvarial sections. Scale bar, 200 μm (upper), 50 μm (lower). (**I**) Quantitative analysis of eroded bone surface per bone surface (EBS/BS, %). (**J**) Representative TRAP staining images of calvarial sections. Scale bar, 200 μm (upper), 50 μm (lower). (**K**) Quantitative analysis of osteoclast surface per bone surface (Oc.S/BS, %). *n* = 6. The selected images reflect typical examples from each group, closely representing the median degree as per statistical analysis. Data are presented as mean ± SD. One-way ANOVA with Tukey’s *post hoc* test. ^**^P < 0.01 *versus* the Sham group. ^#^*P* < 0.05 and ^##^*P* < 0.01 *versus* the TiPs group. *ns*, not statistically significant *versus* the TiPs group

To date, no non-surgical pharmacotherapies have been approved by the Food and Drug Administration (FDA) for arresting aseptic loosening (Goodman et al. 2022; Yu et al. 2024a, b). This highlights the urgent need to identify and develop potential therapeutic agents to prevent and treat periprosthetic osteolysis. Natural phytochemicals have garnered increasing interest in recent years due to their favorable biosafety, remarkable bioactivity, and potential health benefits (Tu et al. 2021; Xu et al. 2024). Osthole (OST), a natural coumarin derivative known chemically as 7-methoxy-8-(3-methyl-2-butenyl) coumarin (Fig. 1A), is abundantly present in many traditional herbal medicines, such as *Cnidium monnieri* (L.) Cusson, *Angelicae Pubescentis* radix, and *Peucedanum ostruthium* (Xu et al. 2024; Kong et al. 2020; Chen et al. 2023). OST has attracted considerable attention due to its diverse pharmacological properties, including anti-inflammation, anti-tumor, antimicrobial, neuroprotective, and osteoprotective effects (Chen et al. 2023; Zafar et al. 2021; Singh and Bhatti 2024). Recent studies have highlighted OST’s potential in promoting osteogenic differentiation and bone formation, positioning it as a promising therapeutic agent for osteoporosis and bone fracture (Zhang et al. 2017; Jin et al. 2021; Wang et al. 2017a, b; Wu et al. 2022; Tang et al. 2010; Gao et al. 2013). Furthermore, emerging evidence suggests that OST may offer protective effects against ER stress-induced cellular damage. For instance, OST has been shown to reduce amyloid plaque deposition and ameliorate cognitive impairment in Alzheimer’s disease by alleviating ER stress (Liu et al. 2023). Additionally, research by Xu and colleagues demonstrated that OST could mitigate porcine circovirus type 2-induced apoptosis by suppressing the ER stress-dependent PERK pathway (Xu et al. 2020, 2023). Notably, a previous study revealed that OST effectively reduced ER stress levels in the calvaria of mice in a model of tricalcium phosphate particle-induced osteolysis, resulting in significant inhibition of osteoclast-mediated bone resorption and inflammatory factor release (Lv et al. 2016). Despite these promising findings, the effects of OST on ER stress-mediated osteogenic impairment in the context of wear particle-induced osteolysis and its underlying mechanisms remain to be fully elucidated.

In this study, we systematically evaluated the pharmacological effects of OST on wear particle-induced osteogenic impairment using a mouse calvarial osteolysis model and an in vitro osteoblast model exposed to particles, with a particular emphasis on investigating its impact on ER stress-mediated apoptosis and osteogenic reduction while trying to elucidate the underlying mechanisms of its action. Our study aims to provide an in-depth understanding of OST’s regulatory role in ER stress and osteogenic function and seeks to elucidate its potential therapeutic benefits in mitigating periprosthetic osteolysis and aseptic loosening. The insights gained from this study are expected to offer a theoretical basis for the development of novel strategies and interventions aimed at arresting aseptic loosening, thereby advancing therapeutic approaches in orthopedic and implant-related diseases.

## Materials and methods

### Particle preparation and characterization

As previously described, TiAl_6_V_4_ particles (TiPs) were sourced from the metallic implants retrieved from patients who underwent revision hip arthroplasty due to aseptic loosening (Yu et al. 2024a, b). All clinical procedures were granted ethical approval by the Clinical Ethics Committee of Nanjing Jinling Hospital, the Affiliated Hospital of Nanjing University Medical School (license no. 2019NZGKL-012), and conformed to the principles of the Helsinki Declaration. Informed consent was obtained from all the participants in this study. The processing and manufacturing of the TiPs were conducted by Professor Zhenzhong Zhang’s team from the College of Materials Science and Engineering of Nanjing University of Technology. Following our established protocol, the implant underwent sterilization and was subsequently transformed into nanoscale wear particles using a fabricated high-vacuum three-electrode direct current system under the following conditions: a vacuum pressure of 10^− 3^ Pa, a gas mixture of 0.04 MPa argon and hydrogen in a 3:2 (v/v) ratio, and a cathode current of 650 A (Yu et al. 2024a, b; Jiang et al. 2018; Deng et al. 2020).

The obtained TiPs were subjected to characterization using Scanning Electron Microscopy (SEM; Regulus-8100, Hitachi, Japan) and Transmission Electron Microscope (TEM; HT7800, Hitachi, Japan), and the average particle diameter of the TiPs measured 51.7 nm (Fig. S1A and S1B) (Yu et al. 2023a, b, 2024a, b). The chemical composition of the TiPs was measured by the energy dispersive spectrometry (EDS) attached to SEM (Fig. S1C). Previous reports have indicated that metal wear particles retrieved from periprosthetic tissues of patients with aseptic loosening typically exhibit nanometer-scale particle sizes (Yu et al. 2024a, b; Doorn et al. 1998). Consequently, the TiPs prepared for this study were well-suited for simulating the wear particles generated from metallic implants within the human body. Endotoxin removal procedures were carried out following previously documented methods (Mediero et al. 2012), ensuring that the endotoxin levels of TiPs were maintained at levels lower than 0.25% EU/ml, as determined using the Pierce LAL Chromogenic Endotoxin Quantitation Kit (ThermoFisher, 88282). The resulting particles were suspended in sterile phosphate-buffered saline (PBS; BOSTER, AR0030) at a stock concentration of 50 mg/ml, followed by autoclaving for 15 min for sterilization. Before utilization, the TiPs suspension was subjected to 20 min of ultrasonication.

### In vivo calvarial osteolysis mouse model

The mouse calvarial osteolysis model was performed following previously established procedures by our research group (Jiang et al. 2018). The C57BL/6J mice (male, 8–10 weeks old) used in this study were purchased from the Model Animal Research Center of Nanjing University (Nanjing, China). All mice were housed in a conventional clean room under constant temperature (22–25 °C), relative humidity (55–60%), and 12 h light-dark cycle conditions, with unlimited access to food and water throughout the experiment period. After one-week adaptive feeding, the mice were randomly divided into four groups (*n* = 12 per group): (1) Group I, Sham-operated mice; (2) Group II, TiPs-treated mice; (3) Group III, mice treated with TiPs receiving the low dose of OST (1 mg/kg per day); (4) Group IV, mice treated with TiPs receiving the high dose of OST (5 mg/kg per day). The dosing regimens for in vivo studies were referred to previous studies (Tang et al. 2010; Jin et al. 2022). For each group, 6 mice were randomly selected for histological analysis, and the remaining 6 mice were used for molecular biological analyses.

Briefly, mice were anesthetized with intraperitoneal phenobarbitone (40 mg/kg, 1%), and the skull was exposed by separating the skin and periosteum. A forty-microliter suspension of TiPs (50 mg/ml) was then placed under the periosteum at the middle suture of the exposed calvaria, followed by suturing of the incision (Yu et al. 2024a, b). Sham-operated mice underwent the same surgical procedure without TiPs treatment. Osthole (OST; Topscience, T2848; Batch No. 114765; Purity: 99.93%) was dissolved in an appropriate amount of dimethyl sulfoxide (DMSO) and then combined with corn oil to prepare an injection (Jin et al. 2022). Mice in groups III and IV were injected subcutaneously over the calvarial surface with the specified dose of OST once daily for 14 consecutive days after surgery, while mice receiving an equivalent volume of corn oil as the control. After two weeks of treatment, all mice were euthanized, and their calvariae and major organs were collected for subsequent analyses (Fig. 1B). Additionally, serum samples were collected for biochemical index detection to evaluate drug safety. All animal experimental procedures were approved by the Animal Ethics Committee of Nanjing Jinling Hospital, the Affiliated Hospital of Nanjing University Medical School (license no. 2019JLHGKJDWLS-073), and adhered to the principles outlined in the National Institutes of Health Guide for the Care and Use of Laboratory Animals.

### Micro-computed tomography (micro-CT) scanning and analysis

Following fixation in 4% paraformaldehyde (PFA) for 24 h, all mouse calvarial samples were subjected to high-resolution micro-CT scanning using a SkyScan 1176 scanner (Bruker, Germany). Scanning parameters included an 18 μm voxel size, with the X-ray source set at 50 kV, and a current of 455 µA. A square region of interest (ROI, 5 mm × 5 mm × 1 mm) centered around the sagittal suture was selected for calculating bone morphometric parameters, specifically bone volume-to-total volume (BV/TV) and bone mineral density (BMD) (Yu et al. 2023a, b). Three-dimensional reconstruction and image analysis were conducted using DataViewer, CTvox, and CTAn software from SkyScan (Wu et al. 2023).

### Histological and immunohistochemical staining

Following micro-CT scanning and analysis, all mouse calvarial samples were decalcified in 10% EDTA solution at 4℃ for 14 days and subsequently embedded in paraffin. The specimens were then sliced into 5-µm-thick sections using an RM2235 microtome (Leica, Germany). Hematoxylin & eosin (H&E) staining was carried out using an H&E staining Kit (Leagene, DH0006) following standard H&E staining procedures. To assess osteoclast activity and the extent of bone erosion, tartrate-resistant acid phosphatase (TRAP) staining was performed using a TRAP staining kit (Wako, 294–67001) following the manufacturer’s protocol. Additionally, collagen staining was conducted using Masson’s trichrome staining with a Masson’s trichrome staining kit (KeyGEN, KGMST-8004) according to the manufacturer’s instructions.

Immunohistochemical (IHC) staining of specific proteins was executed using an IHC kit (KeyGEN, KGOS300) per the manufacturer’s guidelines. The primary antibodies used for the IHC assay were as follows: anti-osteocalcin (OCN) rabbit IgG (Proteintech, 23418-1-AP), anti-Collagen Type I Alpha 1 (COL1α1) rabbit IgG (Boster, PB0981), anti-GRP78 rabbit IgG (Abcam, ab21685), anti-CHOP rabbit IgG (Affinity, AF6277), anti-OCN mouse IgG (Santa Cruz Biotechnology, sc-390877), anti-C-CASP3 rabbit IgG (Cell Signaling Technology, 9664), anti-phospho-PERK (p-PERK) rabbit IgG (Affinity, DF7576), anti-Phospho-eIF2 alpha (p-eIF2α) rabbit IgG (Affinity, AF3087), anti-ATF4 (Affinity, DF6008).

TRAP-stained whole calvariae were observed and imaged using a Nikon SMZ-745 T stereomicroscope (Nikon, Japan), and the percentages of osteolysis area were analyzed within a defined ROI (5 mm × 5 mm) surrounding the sagittal suture (Yu et al. 2023a, b; Sato et al. 2015). Histological sections were observed and photographed centered on the midline suture of the sections using a brightfield microscope (Nikon, Japan) equipped with a Nikon DS-Ri2 camera. The non-osseous tissue adjacent to and continuous with the midline suture was defined as the osteolysis area, positive areas and the number of positive cells were evaluated in three randomly selected visual fields under 40× magnification around the midline suture in each histological Sects. (Xie et al. 2023; Zhu et al. 2023; Wedemeyer et al. 2007).

### Double fluorochrome labeling

For double fluorochrome sequential labeling of newly formed bone, mice were intraperitoneally injected with 20 mg/kg calcein (Sigma-Aldrich, C0875) and 30 mg/kg alizarin red (Sigma-Aldrich, A5533) at 12 and 2 days before euthanasia, respectively (Fig. 1B) (Pflanz et al. 2017; Tanjaya et al. 2018; Wang et al. 2015a, b, c). After fixation with 4% PFA and dehydration with 30% sucrose solution, the calvarial samples were embedded with optimum cutting temperature (OCT) for frozen sectioning. Subsequently, the specimens were cut into 10-µm-thick coronal sections using a cryostat microtome (Leica CM1950, Germany). The interval between calcein and alizarin red labeling was determined using an LSM 980 confocal microscope (Zeiss, Germany), and the mineral apposition rate (MAR, µm/day) was calculated by dividing the mean interval distance by the inter-label time of 10 days.

### In vivo biosafety evaluation

To comprehensively evaluate the in vivo biosafety of TiPs and OST, we conducted serum biochemical analyses and histological examinations (Yu et al. 2024a, b). Briefly, blood samples were obtained via cardiac puncture and allowed to clot at room temperature before being centrifuged at 3000 rpm for 15 min to collect serum. Serum biochemical parameters, including liver function indicators (alanine aminotransferase [ALT], aspartate aminotransferase [AST]) and renal function indicators (blood urea nitrogen [BUN], serum creatinine [SCr]), were measured using an automatic biochemical analyzer (DRI-CHEM NX700i, Fujifilm, Tokyo, Japan). Additionally, major organs (heart, liver, spleen, lung, and kidney) were harvested post-euthanasia to assess systemic toxicity. These organs were fixed in 4% PFA, embedded in paraffin, sectioned, and stained with H&E. The stained sections were examined under a light microscope to assess any histopathological changes indicative of toxicity or adverse effects.

### Western blotting

Total protein was extracted from tissue or cell samples using RIPA lysis buffer (Beyotime, P0013B) supplemented with a protease and phosphatase inhibitor cocktail (Beyotime, P1045), following established protocols (Wang et al. 2017a, b). Protein concentration was determined using a BCA protein concentration detection kit (Solarbio, PC0020). Subsequently, protein extracts were subjected to boiling for 5 min at 100 °C with 5 × SDS loading buffer (Servicebio, G2075). Thirty micrograms of protein from each sample were separated on 12.5% SDS-PAGE gels (Epizyme, PG113) and transferred to polyvinylidene difluoride (PVDF) membrane (0.22 μm, Millipore, ISEQ00010).

To minimize non-specific binding, the transferred membranes were blocked with QuickBlock blocking buffer (Beyotime, P0252FT) for 15 min at room temperature. Subsequently, the membranes were incubated in primary antibody solutions overnight at 4 °C. Following this, the membranes were washed three times with 1×TBST and then incubated with HRP-conjugated secondary antibody solutions for 1 h at room temperature. After another round of cleaning with 1×TBST, the membranes were treated with a chemiluminescence (ECL) developing solution (Vazyme, E411-05), and the protein bands were visualized and analyzed using a Tanon 4200SF imaging analysis system (Tanon, China).

The specific antibodies used for Western blotting were as follows: anti-OCN rabbit IgG (Affinity, DF12303), anti-COL1α1 rabbit IgG (Boster, PB0981), anti-GRP78 rabbit IgG (Abcam, ab21685), anti-CHOP rabbit IgG (Affinity, AF6277), anti-C-CASP3 rabbit IgG (Cell Signaling Technology, 9664), anti-BAX rabbit IgG (Abcam, ab32503), anti-BCL2 rabbit IgG (Abcam, 182858), anti-ALP rabbit IgG (Affinity, DF6225), anti-RUNX2 rabbit IgG (Affinity, AF5186), anti-PERK rabbit IgG (Affinity, AF5304), anti-phospho-PERK rabbit IgG (Affinity, DF7576), anti-eIF2α rabbit IgG (Cell Signaling Technology, 9722), anti-phospho-eIF2α rabbit IgG (Cell Signaling Technology, 3597), anti-ATF4 rabbit IgG (Cell Signaling Technology, 11815), anti-IRE1α rabbit IgG (Abcam, ab37073), anti-phospho-IRE1α rabbit IgG (Abcam, ab48187), anti-XBP1s rabbit IgG (Cell Signaling Technology, 40435), anti-N-ATF6 rabbit IgG (Abcam, ab37149), anti-β-ACTIN rabbit IgG (Affinity, AF7018). Uncropped western blot images are provided in the supplementary material file for full transparency.

### Isolation and culture of primary mouse osteoblasts

Primary mouse osteoblasts were isolated from the calvaria of neonatal mice as previously described (Taylor et al. 2014). Briefly, the calvariae were aseptically dissected, rinsed with sterile PBS, and cut into bone pieces using a sterile ophthalmic scissor. Subsequently, the bone pieces underwent sequential digestion in a mixture of 0.1% collagenase (Sigma-Aldrich, C6885) and 0.125% trypsin (Beyotime, C0207) at 37℃ for 20 min. After digestion, the isolated cells were cultured in alpha-Minimal Essential Medium (α-MEM; Gibco, 12571063) supplemented with 10% fetal bovine serum (FBS; Gibco, 10099141) and 1% Penicillin-Streptomycin (NCM Biotech, C100C5) in a 37 °C incubator with 5% CO_2_. The cells were allowed to proliferate until confluent and then used for subsequent experiments upon reaching passages 3 to 5.

For osteoblast differentiation, the cells were reseeded and cultured in α-MEM supplemented with 10% FBS, 10 nM dexamethasone (Beyotime, ST1254), 50 µg/ml ascorbic acid (Beyotime, ST1434), and 5 mM β-glycerophosphate (Sigma, G9422). The differentiation medium was refreshed every 3 days, and the cells were maintained under these conditions for the specified duration to induce osteogenic differentiation, confirmed by the expression of osteogenic markers and mineralization assays.

### Cell viability assay

Cell viability was assessed using a cell counting kit-8 (CCK-8) assay kit (Dojindo, CK04) following the manufacturer’s instructions. Briefly, primary osteoblasts were seeded in 96-well plates at a density of 8 × 10^3^ cells in 100 µl of culture medium per well. After an overnight incubation to allow cell adhesion, the cells were treated according to the designed experimental protocol for 24 h. Subsequently, 10 µl of CCK-8 solution was added to each well, and the plates were then incubated for 2 h at 37 °C. Finally, the absorbance was measured at 450 nm using a Varioskan LUX multimode microplate reader (Thermo Fisher, USA).

### Flow cytometry analysis

Detection and analysis of apoptosis were performed by flow cytometry using Annexin V-FITC and propidium iodide (PI) staining, as previously described (Yu et al. 2024a, b). Briefly, primary osteoblasts were seeded into 6-well plates at a density of 1 × 10^6^ cells per well and treated according to the designed experimental protocol for 24 h. Following treatment, the cells were harvested and subjected to staining with Annexin V-FITC and PI according to the instructions provided by the manufacturer of the Apoptosis Detection Kit (Solarbio, CA1020). The stained cells were then analyzed using an Attune NxT flow cytometer (Thermo Fisher, USA).

### TUNEL staining

TdT-mediated dUTP nick end-labeling (TUNEL) staining was carried out following the manufacturer’s instructions with a TUNEL Apoptosis Detection kit (Servicebio, G1501). In brief, cells were fixed in 4% PFA for 15 min and then permeabilized with 0.1% Triton X-100 for 5 min. Subsequently, the cells were incubated with a staining solution composed of TdT enzyme, FITC-dUTP, and equilibration buffer for 1 h at 37 °C. Afterward, the cells were counterstained with 4,6-Diamidino-2-phenylindole (DAPI; Solarbio, C0060) to label the nuclei. TUNEL-positive cells, which emit green fluorescence, were visualized and captured using a confocal fluorescence microscope (LSM 980, ZEISS, Germany).

### Immunofluorescence staining

To evaluate the levels of ER stress and apoptosis in osteoblasts, fixed samples were treated with 0.1% Triton X-100 for permeabilization and blocked with 5% bovine serum albumin (BSA; Beyotime, ST023). Following this, the samples were incubated overnight at 4 °C with primary antibodies specific to GRP78 (Affinity, AF5366), CHOP (Affinity, AF6277), C-CASP3 (Cell Signaling Technology, 9664), or OCN (Santa Cruz Biotechnology, sc-390877), and then incubated with appropriate secondary antibodies labeled with Alexa Fluor 488 or Alexa Fluor 546 (Invitrogen, USA) for 1 h at room temperature (Yu et al. 2024a, b). For F-actin cytoskeleton staining of osteoblasts, the prepared cells were directly stained with DyLight™ 554 Phalloidin (Cell Signaling Technology, 13054) for 1 h at 37 °C. Finally, after staining the nuclei with DAPI (Solarbio, C0060), the stained tissue and cells were observed and imaged using a confocal fluorescence microscope (LSM 980, ZEISS, Germany).

### Alkaline phosphatase (ALP) staining

For ALP staining, primary osteoblasts were subjected to osteogenic induction for 7 days. After the induction period, the cells were fixed in 4% PFA for 15 min and then rinsed three times with PBS. Subsequently, the fixed cells were stained using a BCIP/NBT ALP Color Development Kit (Beyotime, C3206) according to the manufacturer’s instructions. The stained cells were observed under a light microscope, and images were captured for further analysis. Dark purple or blue coloration indicated areas of high ALP activity, signifying osteoblast differentiation and maturation.

### Alizarin red S (ARS) staining

For ARS staining, primary osteoblasts were subjected to osteogenic induction for 14 days. After the induction period, the cells were fixed in 4% PFA for 15 min and then rinsed with double-distilled H_2_O. Following fixation and rinsing, the cells were stained with an ARS Staining Kit (Beyotime, C0148S) to identify mineralized nodules, as per the manufacturer’s instructions. The stained cells were then observed and imaged under a light microscope to capture the red coloration indicative of calcium deposits. For quantitative analysis, the bound ARS dye was eluted with 10% cetylpyridinium chloride, and the absorbance was measured at 562 nm using a Varioskan LUX multimode microplate reader (Thermo Fisher, USA).

### RNA sequencing (RNA-seq) analysis

Total RNA was extracted from primary osteoblasts treated with or without TiPs at a concentration of 50 µg/ml for 24 h (*n* = 3). RNA sequencing and subsequent analyses were performed by Huada Gene Company to obtain the Fragments Per Kilobase of transcript per Million mapped reads (FPKM) values for all genes. Differentially expressed genes (DEGs) were analyzed using the limma package within the R software environment. A significance threshold of *p* < 0.05, and fold changes > 1.5 were applied for identifying DEGs.

### PCR analysis of XBP1 mRNA

To amplify both the spliced and unspliced XBP1 mRNA, specific primers for XBP1 were used as described previously (Forward: 5’-ACACGCTTGGGAATGGACAC-3’, reverse: 5’-CCATGGGAAGATGTTCTGGG-3’) (Yu et al. 2024a, b; An et al. 2024). Briefly, complementary DNA (cDNA) was synthesized from extracted RNA and then subjected to PCR amplification using the XBP1-specific primers. This process generated a 171 bp fragment for unspliced XBP1 (XBP1u) and a 145 bp fragment for spliced XBP1 (XBP1s). The β-actin band served as a reference to ensure equal loading and quality of the RNA samples. The PCR products were separated by electrophoresis on a 3% agarose gel stained with ethidium bromide, followed by visualization under UV light using a gel imaging system (Tanon 2500). To ensure the reliability and accuracy of the PCR results, β-actin mRNA was used as a control for the PCR process (Forward: 5’-GGCTGTATTCCCCTCCATCG-3’, reverse: 5’-CCAGTTGGTAACAATGCCATGT-3’). For full transparency, uncropped agarose gel images are provided in the supplementary material file.

### Molecular docking

The chemical structure of OST was obtained from PubChem in .sdf format (PubChem CID: 10228) and converted to .pdb format using Chem 3D 20.0, and then subjected to energy minimization using MM2 force field. The X-ray crystal structure of PERK complexed with GSK2606414 (PDB ID: 4G31) was chosen as the core target protein (Axten et al. 2012). Before initiating the docking simulation, the co-crystal ligand and structural water molecules were removed from the protein structure. The prepared ligand and protein structures were then imported into AutoDock Tools 1.5.7, where hydrogen atoms were added, charges were calculated, and the structures were converted to .pdbqt format. Finally, Molecular docking was performed using AutoDock Vina 1.1.2, and the results were visualized using PyMOL 2.5.4.

### Statistical analysis

Statistical analyses were performed using GraphPad Prism software (Version 8.0.2), and data are expressed as mean ± standard deviation (SD). The Shapiro-Wilk test were performed to assess the normality of data distribution, and the F test (two groups) or Brown-Forsythe test (multiple groups) for determining homogeneity of variance. For the data not conforming to a normal distribution, nonparametric tests were used, either Mann-Whitney U test (two groups) or Kruskal-Wallis test (multiple groups). For the data conforming to a normal distribution, the following statistical analyses were performed. Two-group comparisons: Unpaired two-tailed Student’s *t*-test was performed (with Welch’s correction applied in cases of unequal variances); Multiple-group comparisons: One-way analysis of variance (ANOVA) followed by Tukey’s *post hoc* tests (for equal variances) or Brown-Forsythe and Welch ANOVA tests followed by Tamhane T2 *post hoc* tests (for unequal variances). A significance level of *p* < 0.05 was considered statistically significant, while *p* < 0.01 was considered strongly significant.

## Results

### OST treatment attenuated TiPs-induced osteolysis in a mouse calvarial model

To investigate the effects of Osthole (OST) on TiAl_6_V_4_ particle (TiPs)-induced osteolysis in a mouse calvarial model, micro-CT scanning, H&E staining, and TRAP staining were performed. Gross observations of TRAP-stained whole calvaria indicated that OST alleviated TiPs-induced bone destruction in a dose-dependent manner (Fig. 1C and D). The micro-CT results corroborated these gross observations, demonstrating a significant reduction in bone loss with OST treatment (Fig. 1E and F). Quantitative analysis of bone morphometric parameters revealed increased bone volume-to-total volume (BV/TV) and bone mineral density (BMD) in OST-treated groups compared to the TiPs group (Fig. 1G). Histological evidence further supported these findings. As shown in Fig. 1H and I, the results of H&E staining showed that OST-treated groups exhibited better-preserved bone architecture and less bone erosion compared to the TiPs group. Moreover, TRAP staining revealed that OST partially inhibited the excessive osteoclast activation induced by TiPs, which is consistent with the previous findings reported by Lv et al. (Lv et al. 2016).

To further assess the biosafety of OST in vivo, we monitored changes in mouse body weight, conducted serum biochemical assays to evaluate liver and kidney function, and performed morphological and histopathological analyses of vital organs, including heart, liver, spleen, lung, and kidney. As shown in Fig. S2A, OST treatment did not adversely affect mouse body weight throughout the experimental period. Moreover, serum biochemical markers of liver function, such as alanine aminotransferase (ALT) and aspartate aminotransferase (AST), as well as kidney function indicators, including blood urea nitrogen (BUN) and serum creatinine (SCr), exhibited no significant abnormalities (Fig. S2B). In addition, the size and surface characteristics of vital organs remained within normal ranges across all groups, and histological examination of H&E-stained tissue sections revealed no pathological changes (Fig. S2C and S2D). These findings suggested that OST appears to possess a favorable biosafety profile, supporting its potential for further therapeutic applications.

### OST treatment mitigated TiPs-induced osteogenic impairment in vivo

Wear particle-induced osteogenic impairment is an important cause of periprosthetic osteolysis and subsequent aseptic loosening (O’Neill et al. 2013; Zhang et al. 2020). Previous studies have demonstrated that OST exhibits potent osteogenic and bone-protective properties, highlighting its potential therapeutic applications in bone-related disorders (Xu et al. 2024; Chen et al. 2023). However, the specific effects of OST on wear particle-induced osteogenic impairment remain largely unexplored. Thus, we further investigated the influence of OST on osteogenesis in mouse calvaria.

**Fig. 2:**
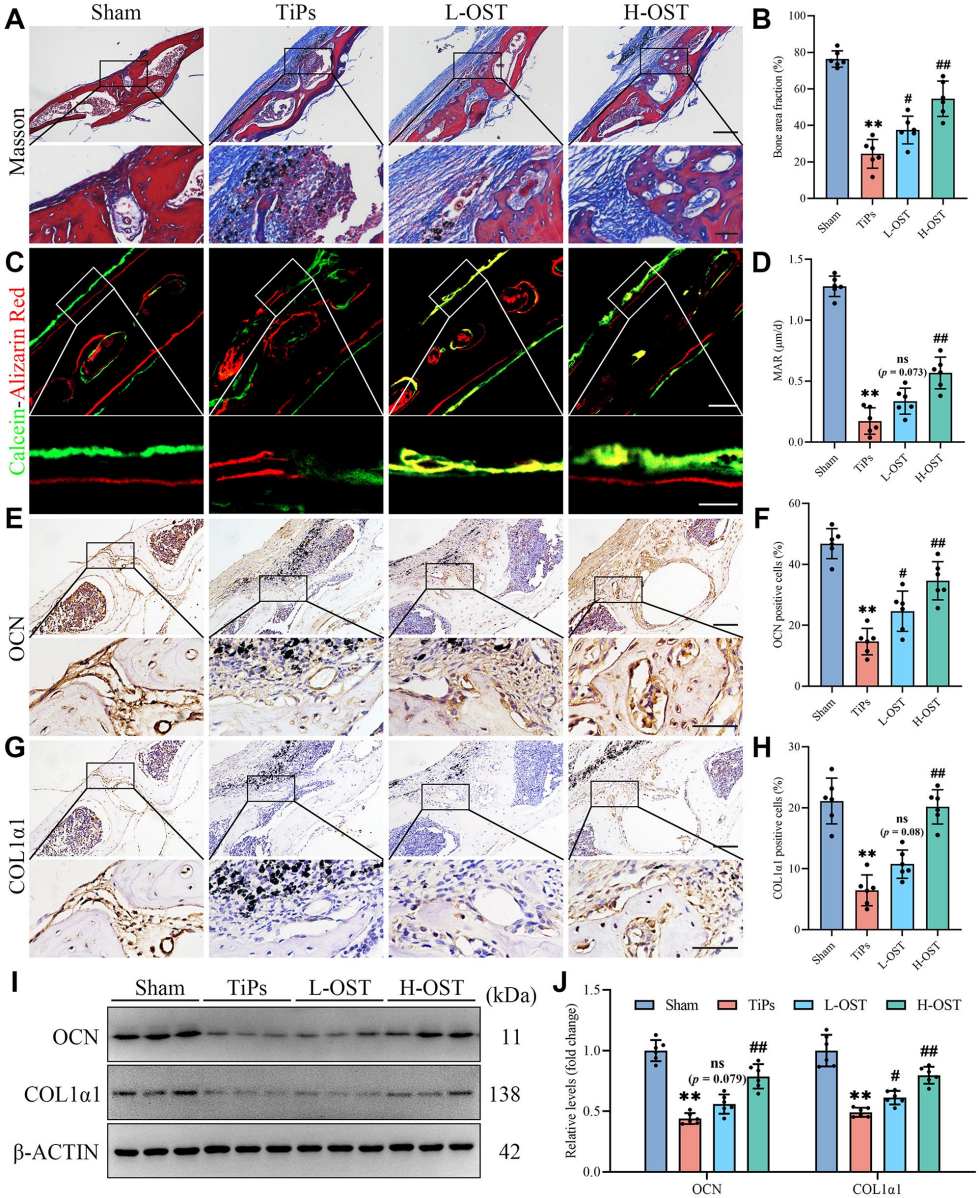
OST treatment attenuated TiPs-induced osteogenesis inhibition in vivo. (**A**) Representative Masson’s trichrome staining images of calvarial sections. Scale bar, 200 μm (upper), 50 μm (lower). (**B**) Quantitative analysis of new bone area fraction (%) determined by Masson’s trichrome staining. (**C**) Representative images of calcein (green) and alizarin red (red) double labeling with a 10-day interval. Scale bar, 50 μm (upper), 20 μm (lower). (**D**) Quantitative analysis of average periosteum mineral apposition rates (MAR, µm/d). (**E-F**) Representative images and quantitative analysis of immunohistochemical (IHC) staining for osteocalcin (OCN). Scale bar, 100 μm (upper), 50 μm (lower). (**G-H**) Representative images and quantitative analysis of IHC staining for collagen type I alpha 1 (COL1α1). Scale bar, 100 μm (upper), 50 μm (lower). (**I-J**) Western blot analysis of the expression of osteogenic markers (OCN, COL1α1) in calvarial bone tissue samples from each group. *n* = 6. The selected images reflect typical examples from each group, closely representing the median degree as per statistical analysis. Data are presented as mean ± SD. One-way ANOVA with Tukey’s *post hoc* test. The relative COL1α1 protein levels were analyzed using Brown-Forsythe and Welch ANOVA tests followed by Tamhane T2 *post hoc* tests. ^**^P < 0.01 *versus* the Sham group. ^#^*P* < 0.05 and ^##^*P* < 0.01 *versus* the TiPs group. *ns*, not statistically significant *versus* the TiPs group

As shown in Fig. 2A and B, Masson’s trichrome staining revealed an increased presence of newly formed bone matrix in OST-treated groups compared to the TiPs group, suggesting that OST treatment effectively promotes bone regeneration and mitigates the adverse effects of wear particles. Moreover, calcein and alizarin red double labeling provided additional evidence of OST’s efficacy in enhancing bone formation, as reflected by an increased mineral apposition rate (MAR) in OST-treated groups (Fig. 2C and D). In addition, immunohistochemical (IHC) analysis was also performed to evaluate the expression of pivotal osteogenic markers, such as osteocalcin (OCN) and Collagen Type I Alpha 1 (COL1α1). As shown in Fig. 2E-H, the IHC results revealed that OST treatment upregulated the expression of OCN and COL1α1, further supporting its role in enhancing osteogenesis. Consistent with the IHC findings, Western blot analysis corroborated the increased expression of OCN and COL1α1 in OST-treated groups compared to the TiPs group (Fig. 2I and J). In conclusion, these findings indicated that OST treatment effectively mitigated TiPs-induced osteogenic impairment in vivo.

### OST treatment reduced ER stress-mediated apoptosis in osteoblasts exposed to TiPs

**Fig. 3:**
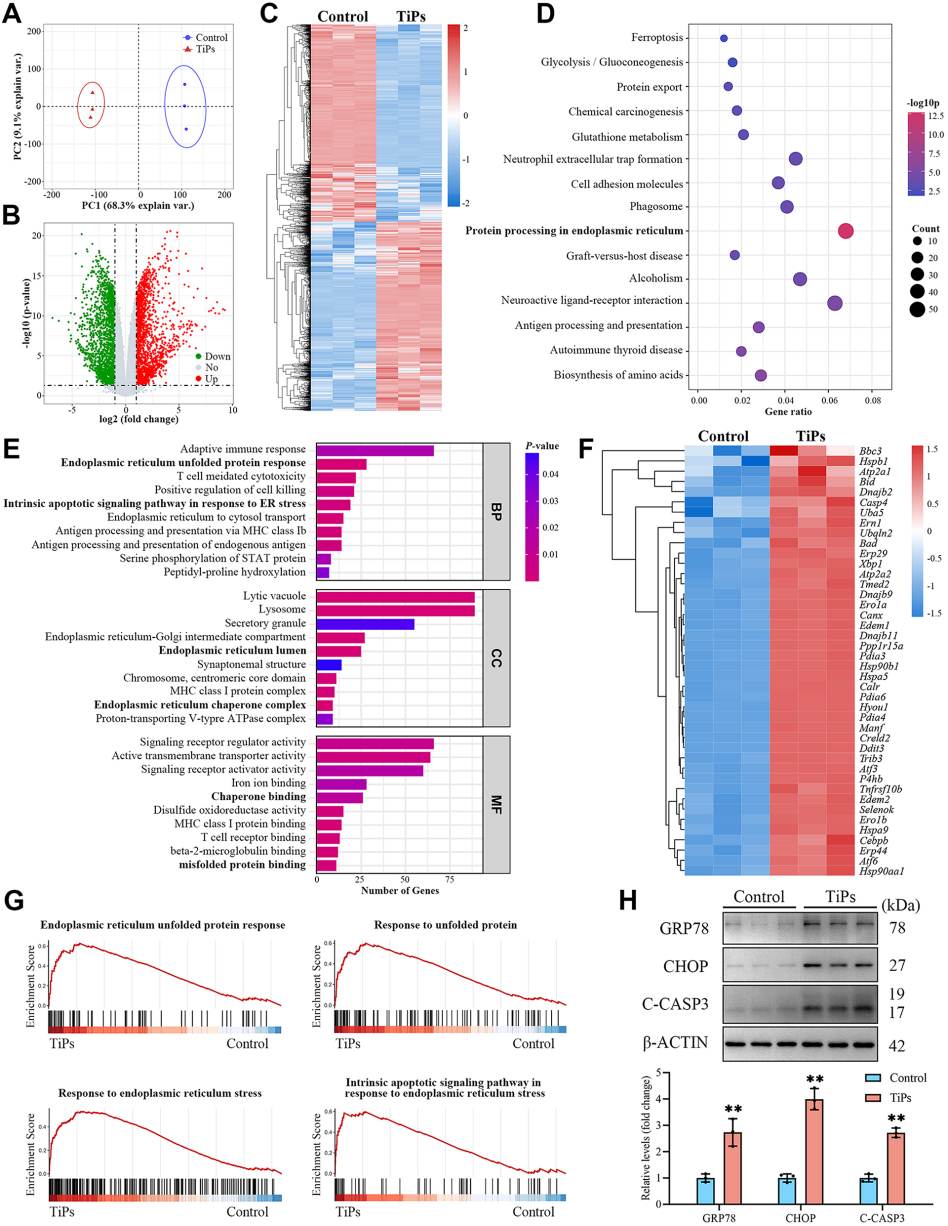
ER stress is involved in mediating TiPs-induced osteoblast apoptosis. (**A**) Principal component analysis (PCA) plot of RNA sequencing (RNA-seq) data obtained from primary osteoblasts treated with or without TiPs (50 µg/ml) for 24 h. (**B**) Volcano plot illustrating upregulated (red) and downregulated (green) genes in the TiPs group compared to the Control group. (**C**) Heat map of the genes differentially expressed between the TiPs group and the Control group. (**D**) KEGG enrichment analysis of differentially expressed genes (DEGs). (**E**) Gene Ontology (GO) analysis of biological processes (BP), cellular components (CC), and molecular functions (MF) of DEGs. (**F**) Heat map of the ER stress-associated DEGs in the TiPs group *versus* the Control group. (**G**) Gene set enrichment analysis (GSEA) showing the ER unfolded protein response, response to unfolded protein, response to ER stress, and intrinsic apoptotic signaling pathway in response to ER stress GO items enrichment in the TiPs group *versus* the Control group. (**H**) Western blot analysis of the expression of ER chaperone GRP78 and ER stress-related pro-apoptotic transcription factor CHOP, as well as the apoptosis executor C-CASP3 in osteoblasts treated with or without TiPs (50 µg/ml) for 24 h. *n* = 3. Data are presented as mean ± SD. Unpaired two-tailed Student’s *t* test. ^**^P < 0.01 *versus* the Control group

Increased osteoblast apoptosis triggered by wear particles leads to decreased bone formation around the implant, as previous studies have established that ER stress plays a critical role in mediating wear particle-induced osteoblast apoptosis (Yu et al. 2023a, b, 2024a, b; Yang et al. 2019). To further substantiate the involvement of ER stress in particle-induced osteogenic impairment, we conducted a comprehensive analysis of differentially expressed genes (DEGs) in osteoblasts treated with or without TiPs using RNA sequencing (RNA-seq). The principal component analysis (PCA) of the RNA-seq data revealed distinct clustering of TiPs-treated osteoblasts compared to untreated controls, indicating significant changes in gene expression profiles due to TiPs exposure (Fig. 3A). As shown in the volcano plot and heatmap in Fig. 3B and C, respectively, a total of 17,110 genes were identified, of which 2327 were differentially expressed. Among these DEGs, 1114 genes were upregulated and 1213 genes were downregulated in response to TiPs treatment. Kyoto Encyclopedia of Genes and Genomes (KEGG) and Gene Ontology (GO) enrichment analyses indicated that these DEGs were primarily involved in pathways and biological processes related to ER protein processing and unfolded protein response (UPR) (Fig. 3D and E). Furthermore, the heatmap in Fig. 3F demonstrated that the expression of ER stress-related genes, such as *Hspa5*, *Ddit3*, *Ero1a*, and *Pdia4*, was significantly upregulated in osteoblasts treated with TiPs. As shown in Fig. 3G, Gene Set Enrichment Analysis (GSEA) further supported these findings by identifying that the gene sets related to ER stress and UPR were enriched in TiPs-treated osteoblasts. Notably, GSEA revealed specific upregulation of pathways associated with ER stress-mediated apoptosis, underscoring the severe impact of TiPs on cellular homeostasis and viability.

To validate the RNA-seq findings, we assessed the protein expression levels of ER stress and apoptosis markers in osteoblasts. Western blot analysis demonstrated an evident increase in the levels of cleaved caspase-3 (C-CASP3), a key indicator of apoptosis, along with upregulated expression of ER stress markers, including the ER chaperone GRP78 and the pro-apoptotic transcription factor CHOP, in osteoblasts treated with TiPs (Fig. 3H). Additionally, we observed that as the concentration of TiPs increased, the levels of ER stress and apoptosis in osteoblasts correspondingly escalated (Fig. S3A and S3B). These findings provided compelling evidence for the importance of ER stress as a major contributing factor to wear particle-induced osteogenic impairment.

**Fig. 4:**
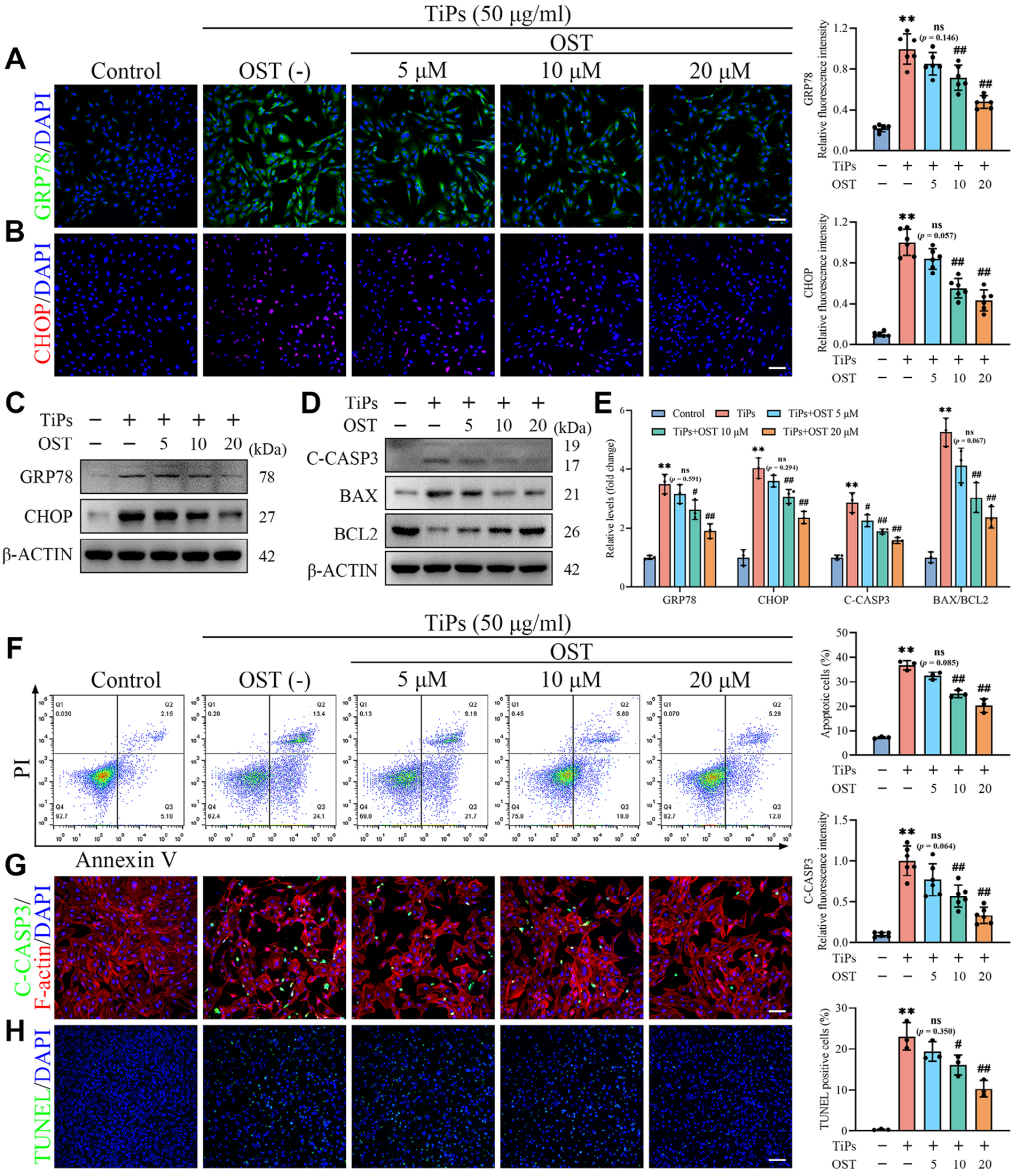
OST treatment attenuated ER stress-mediated apoptosis in osteoblasts exposed to TiPs in vitro. (**A**) Representative images and quantitative analysis of GRP78 immunofluorescence staining (green) in osteoblasts exposed to TiPs (50 µg/ml) following co-treatment with various concentrations of OST (0, 5, 10, and 20 µM) for 24 h. Scale bar, 100 μm. *n* = 6. (**B**) Representative images and quantitative analysis of CHOP immunofluorescence staining (red) in osteoblasts treated as intended. Scale bar, 100 μm. *n* = 6. (**C-E**) Western blot analysis of GRP78, CHOP, C-CASP3, BAX, and BCL2 expression in osteoblasts treated as intended. *n* = 3. (**F**) Flow cytometry analysis of apoptosis levels in osteoblasts treated as intended, using Annexin V/PI staining for detection. *n* = 3. (**G**) Representative images and quantitative analysis of C-CASP3 immunofluorescence staining (green) in osteoblasts treated as intended, with F-actin labeled in red using phalloidin. Scale bar, 100 μm. *n* = 6. (**H**) Representative images and quantitative analysis of TUNEL staining in osteoblasts treated as intended. *n* = 3. Scale bar, 100 μm. The selected images reflect typical examples from each group, closely representing the median degree as per statistical analysis. Data are presented as mean ± SD. One-way ANOVA with Tukey’s *post hoc* test. ^**^P < 0.01 *versus* the Control group. ^#^*P* < 0.05 and ^##^*P* < 0.01 *versus* the TiPs group. *ns*, not statistically significant *versus* the TiPs group

In recent years, increasing evidence suggested that OST may offer protective effects against ER stress-induced cellular damage (Liu et al. 2023; Xu et al. 2020, 2023; Lv et al. 2016). Consequently, we further investigated the effects of OST on osteoblasts exposed to TiPs in vitro, focusing on its potential to alleviate ER stress and apoptosis. As shown in Fig. 4A and B, immunofluorescence results indicated that TiPs treatment significantly increased the expression of ER stress markers GRP78 and CHOP in osteoblasts. In contrast, OST treatment downregulated the expression of these markers in a dose-dependent manner. Western blot analysis consistently confirmed these findings, demonstrating that OST treatment can effectively mitigate the ER stress response induced by TiPs exposure (Fig. 4C and E).

Subsequently, we assessed the effects of OST on osteoblast apoptosis induced by TiPs in vitro. Cell viability assays indicated that OST rescued the decline in cell viability caused by TiPs in a dose-dependent manner, with the effective concentrations being within a safe range (Fig. S4A-C). As shown in Fig. S4D and S4E, the CC50 of OST was determined to be approximately 124.8 µM, while the IC50 for inhibiting TiPs-induced cytotoxicity in osteoblasts was approximately 18.92 µM, further indicating a favorable therapeutic index with effective concentrations well below cytotoxic levels. Western blot results showed that TiPs exposure significantly increased C-CASP3 expression level, whereas OST treatment mitigated these changes induced by TiPs. Moreover, OST treatment reduced the expression of the pro-apoptotic marker BAX while simultaneously promoting the expression of the anti-apoptotic marker BCL2, indicating a shift in the balance towards cell survival (Fig. 4D and E). Flow cytometry analysis using Annexin V/PI staining supported these findings by revealing a decrease in the apoptosis rate in osteoblasts exposed to TiPs following OST treatment (Fig. 4F and S4F). Immunofluorescence staining showed a decrease in C-CASP3 levels, further demonstrating reduced apoptosis in osteoblasts treated with OST under TiPs exposure conditions (Fig. 4G). Concurrently, there was a notable improvement in cytoskeletal integrity, as revealed by phalloidin staining of actin filaments. Upon TiPs exposure, osteoblasts typically exhibited classic apoptotic features, including cell shrinkage, membrane blebbing, and loss of contact with neighboring cells (Chen et al. 2024). These morphological alterations are closely associated with cytoskeletal disorganization, which plays a crucial role in apoptosis progression (Ndozangue-Touriguine et al. 2008). However, compared with osteoblasts exposed to TiPs alone, the actin cytoskeleton in OST-treated osteoblasts exhibited a more organized and intact actin network, with obvious improvements in cell morphology (Fig. 4G). Additionally, TdT-mediated dUTP nick end-labeling (TUNEL) staining provided further evidence of decreased apoptotic cell death in OST-treated osteoblasts compared to those exposed to TiPs alone (Fig. 4H).

**Fig. 5:**
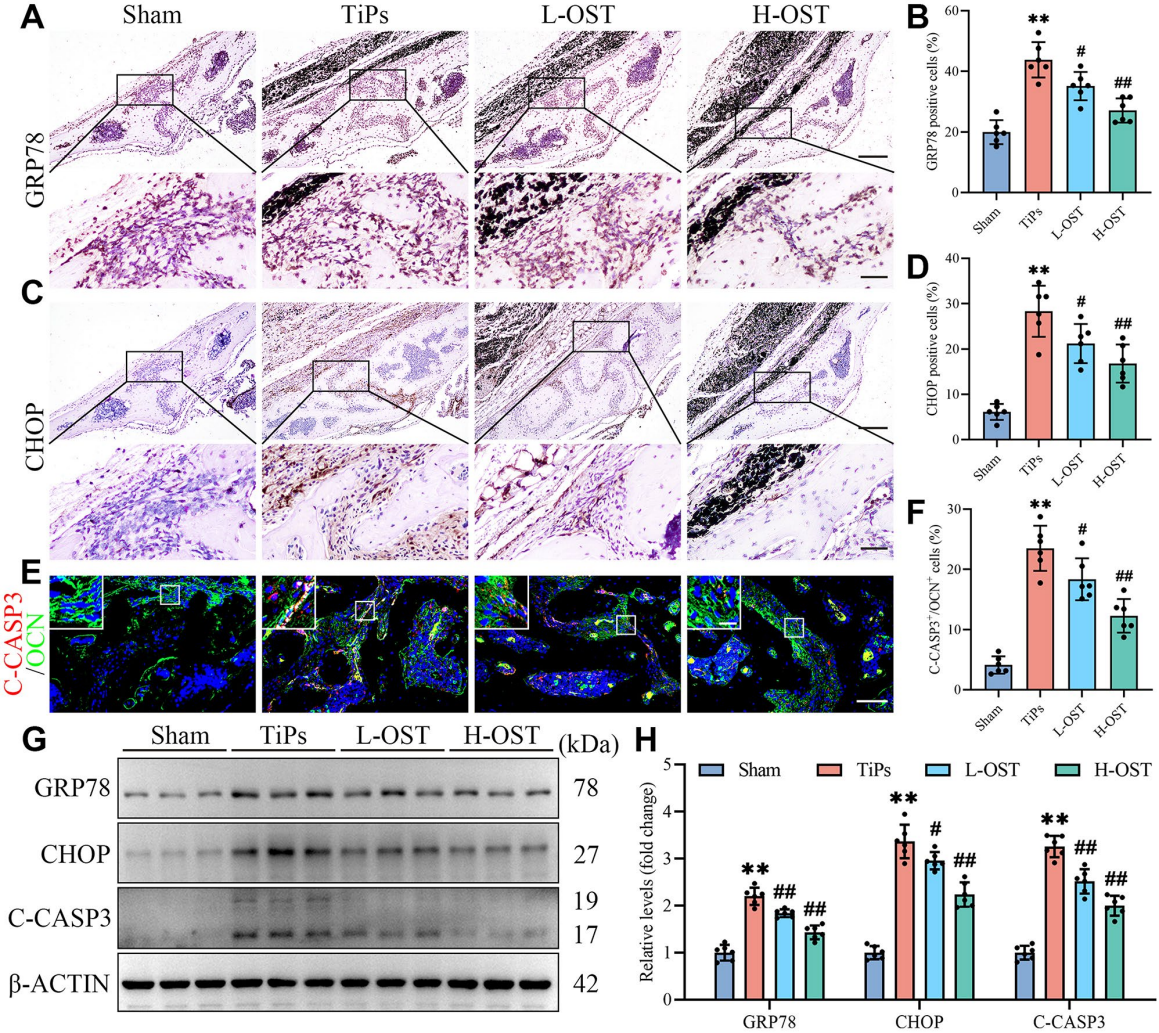
OST treatment mitigated ER stress-mediated osteoblast apoptosis in the TiPs-induced mouse calvarial osteolysis model. (**A-B**) Representative images and quantitative analysis of IHC staining for GRP78. Scale bar, 200 μm (upper), 50 μm (lower). (**C-D**) Representative images and quantitative analysis of IHC staining for CHOP. Scale bar, 200 μm (upper), 50 μm (lower). (**E-F**) Representative images and quantitative analysis of immunofluorescence double labeling for C-CASP3 (red) and OCN (green). Scale bar, 100 μm. The inset in the upper-left corner shows the magnification of the white boxed region. Scale bar, 20 μm. (**G-H**) Western blot analysis of GRP78, CHOP, and C-CASP3 expression in calvarial bone tissue samples from each group. *n* = 6. The selected images reflect typical examples from each group, closely representing the median degree as per statistical analysis. Data are presented as mean ± SD. One-way ANOVA with Tukey’s *post hoc* test. ^**^P < 0.01 *versus* the Sham group. ^#^*P* < 0.05 and ^##^*P* < 0.01 *versus* the TiPs group

Furthermore, we conducted in vivo immunostaining and Western blot analyses to evaluate the effects of OST treatment on ER stress-mediated apoptosis in osteoblasts exposed to TiPs within mouse calvaria. As shown in Fig. 5A-D, the IHC results revealed a significant reduction in GRP78 and CHOP expression levels in the OST-treated group compared to the TiPs group, indicating that OST effectively mitigated TiPs-induced ER stress in vivo. In addition, double immunofluorescence staining showed a notable decrease in co-localization of OCN with C-CASP3 in the OST-treated group, suggesting that OST attenuated osteoblast apoptosis induced by TiPs in vivo (Fig. 5E and F). Western blot analysis further corroborated these results, showing downregulation of GRP78, CHOP, and C-CASP3 protein levels in the OST-treated group compared to the TiPs group (Fig. 5G and H). Together, these findings provided robust evidence that OST exerts protective effects against ER stress-mediated apoptosis in osteoblasts subjected to TiPs, highlighting its potential as a therapeutic agent for mitigating wear particle-induced osteolysis.

### OST treatment rescued TiPs-induced osteogenic inhibition in vitro

**Fig. 6:**
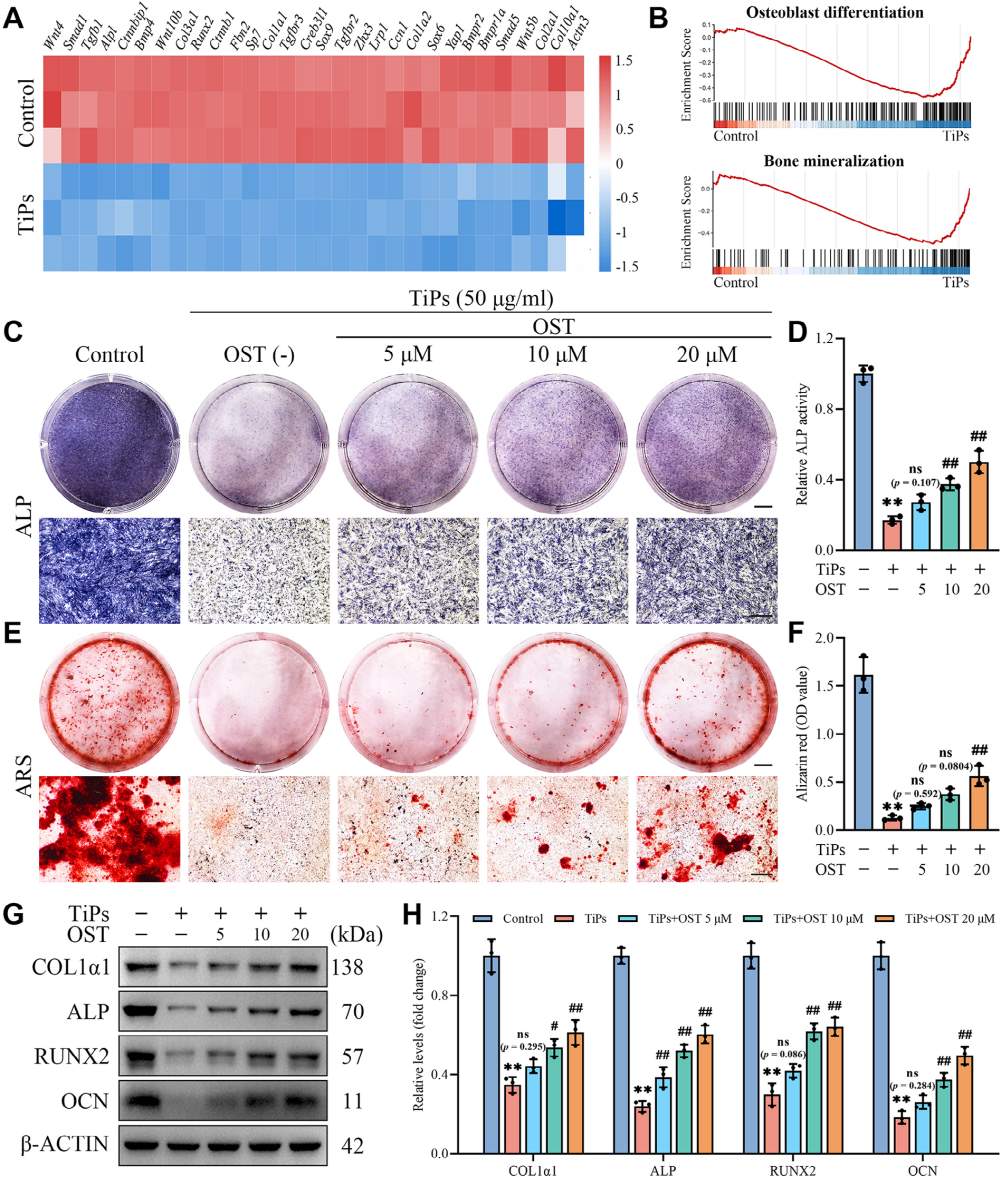
OST treatment improved the impaired osteogenic potential of osteoblasts upon TiPs exposure in vitro. (**A**) Heat map of the osteogenesis-related DEGs in the TiPs group *versus* the Control group. (**B**) GSEA illustrating the enrichment of osteoblast differentiation and bone mineralization GO items, which are negatively regulated in the TiPs group compared to the Control group. (**C**) Representative images of alkaline phosphatase (ALP) staining of osteoblasts treated as intended following 7 days of osteogenic induction. Scale bar, 5 mm (upper), 500 μm (lower). (**D**) Quantitative analysis of relative ALP activity. (**E**) Representative images of alizarin red S (ARS) staining of osteoblasts treated as intended following 14 days of osteogenic induction. Scale bar, 5 mm (upper), 500 μm (lower). (**F**) Quantitative analysis of ARS staining. (**G-H**) Western blot analysis of the expression of osteogenesis-related proteins (COL1α1, ALP, RUNX2, and OCN) in osteoblasts treated as intended following 3 days of osteogenic induction. *n* = 3. The selected images reflect typical examples from each group, closely representing the median degree as per statistical analysis. Data are presented as mean ± SD. One-way ANOVA with Tukey’s *post hoc* test. ^**^P < 0.01 *versus* the Control group. ^#^*P* < 0.05 and ^##^*P* < 0.01 *versus* the TiPs group. *ns*, not statistically significant *versus* the TiPs group

Based on RNA-seq analysis, the heatmap shown in Fig. 6A illustrated a significant downregulation of osteogenesis-related genes, such as *Alpl*, *Sp7*, *Col1a1*, and *Tgfb1*, in osteoblasts treated with TiPs compared to untreated controls. Moreover, GSEA analysis indicated that TiPs exposure negatively regulated the GO items associated with osteoblast differentiation and bone mineralization, further supporting the detrimental effects of TiPs exposure on osteogenic potential (Fig. 6B). Previous studies have reported that OST possesses the ability to promote osteoblast differentiation and mineralization, implying its potential to counteract the detrimental effects induced by TiPs on osteoblast function (Xu et al. 2024; Chen et al. 2023).

To investigate the effects of OST on the osteogenic capacity of osteoblasts exposed to TiPs, we performed alkaline phosphatase (ALP) staining and alizarin red S (ARS) staining to evaluate the levels of osteoblast differentiation and mineralization, respectively. As shown in Fig. 6C and D, ALP staining results revealed that TiPs exposure significantly decreased ALP activity in osteoblasts, while this reduction was effectively rescued by OST treatment, suggesting a restoration of osteogenic differentiation. Similarly, ARS staining results showed that TiPs exposure led to a marked reduction in mineralized matrix formation, whereas OST treatment partially restored mineralization levels, as evidenced by the increased presence of red-stained mineralization nodules in OST-treated groups (Fig. 6E and F). To further validate the rescuing effects of OST on TiPs-induced osteogenic inhibition, Western blot analysis was performed to detect the expression levels of four crucial osteogenesis-related proteins (COL1α1, ALP, RUNX2, OCN). As shown in Fig. 6G and H, the expression levels of these osteogenic markers were evidently reduced in osteoblasts exposed to TiPs compared to untreated controls. However, OST treatment effectively restored their expression levels, reinforcing the notion that OST can mitigate the adverse effects of wear particles on osteoblast function.

### OST treatment inhibited activation of the PERK signaling cascade in osteoblasts exposed to TiPs

**Fig. 7:**
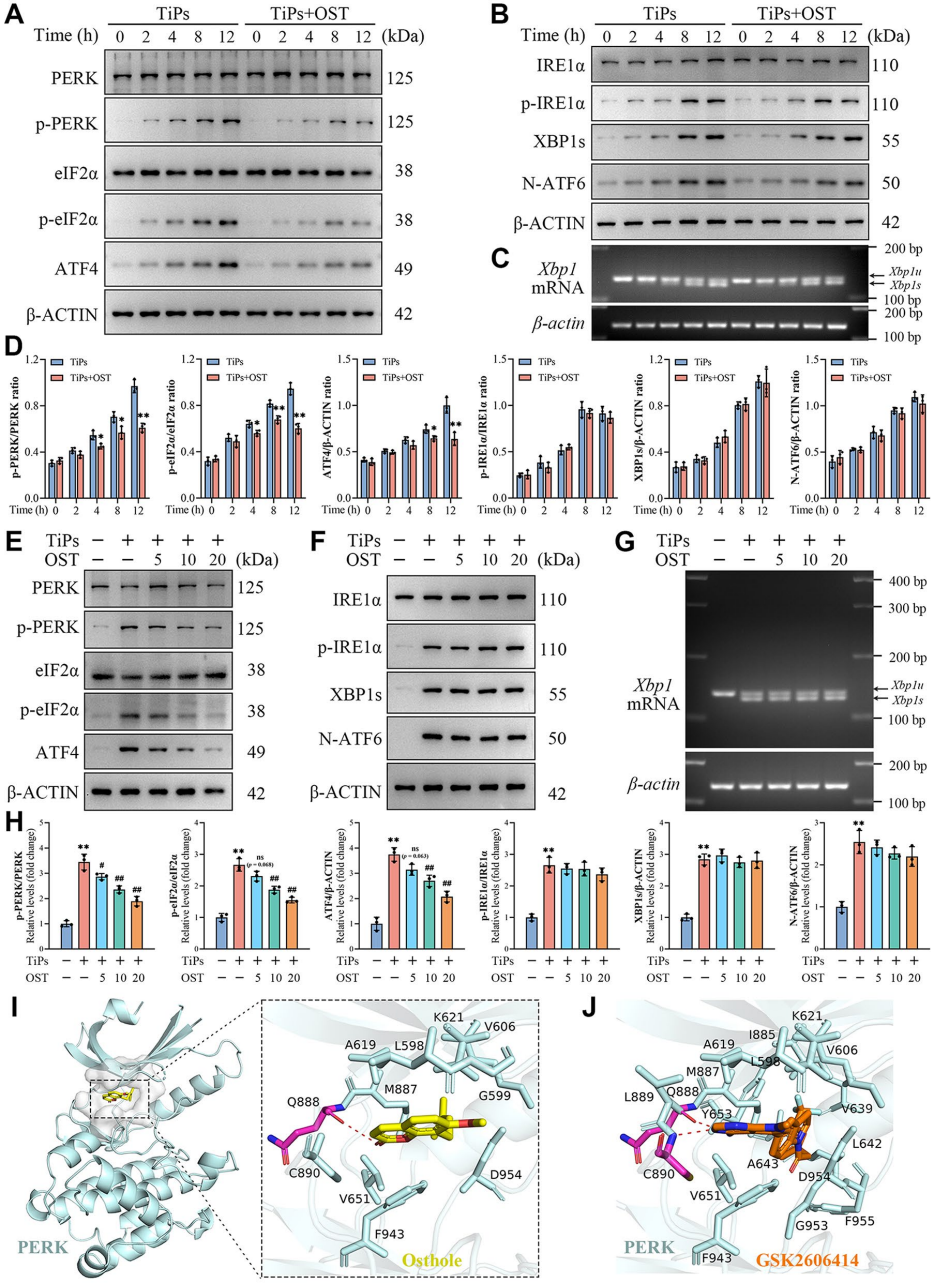
OST treatment specifically suppressed the activation of the PERK signaling cascade in osteoblasts upon TiPs exposure in vitro. (**A**) Western blots showing the protein levels of PERK, p-PERK, eIF2α, p-eIF2α, and ATF4 in osteoblasts upon TiPs exposure (50 µg/ml) following treatment with or without OST (20 µM) for 0, 2, 4, 8, and 12 h. (**B**) Western blots showing the protein levels of IRE1α, p-IRE1α, XBP1s, and N-ATF6 in osteoblasts treated as intended. (**C**) Agarose gel electrophoresis of XBP1 PCR products to detect the splicing of XBP1 mRNA in osteoblasts treated as intended, with *β-actin* as the loading control. (**D**) Quantitative analysis of the western blot results of (A) and (B). *n* = 3. Data are presented as mean ± SD. Unpaired two-tailed Student’s *t* test. ^*^P < 0.05 and ^**^P < 0.01 *versus* the Control group. (**E**) Western blots showing the protein levels of PERK, p-PERK, eIF2α, p-eIF2α, and ATF4 in osteoblasts upon TiPs exposure (50 µg/ml) following co-treatment with various concentrations of OST (0, 5, 10, and 20 µM) for 12 h. (**F**) Western blots showing the protein levels of IRE1α, p-IRE1α, XBP1s, and N-ATF6 in osteoblasts treated as intended. (**G**) Agarose gel electrophoresis of XBP1 PCR products to detect the splicing of XBP1 mRNA in osteoblasts treated as intended, with *β-actin* as the loading control. (**H**) Quantitative analysis of the western blot results of (E) and (F). *n* = 3. Data are presented as mean ± SD. One-way ANOVA with Tukey’s *post hoc* test. ^**^P < 0.01 *versus* the Control group. ^#^*P* < 0.05 and ^##^*P* < 0.01 *versus* the TiPs group. *ns*, not statistically significant *versus* the TiPs group. (**I-J**) Molecular docking poses of OST and GSK2606414 within the nucleotide-binding site of the PERK kinase domain, generated using AutoDock and visualized with PyMOL. Dashed red lines represent hydrogen bonding interactions

The findings presented above suggested that OST treatment might alleviate TiPs-induced osteogenic impairment in osteoblasts by mitigating ER stress. To further elucidate the underlying mechanisms, we examined the impact of OST on the activation of unfolded protein response (UPR) pathways triggered by TiPs. As shown in Fig. 7A-D, time course experiments were performed to assess the activation of each UPR branch driven by PERK, IRE1α, and ATF6 in osteoblasts exposed to TiPs with or without OST treatment. Our results revealed a clear time-dependent activation manner of all three UPR branches in response to TiPs exposure. Specifically, the PERK pathway showed marked upregulation, indicated by increased phosphorylation of PERK and its downstream target eIF2α, as well as elevated expression of ATF4 (Fig. 7A and D). Similarly, the IRE1α pathway also demonstrated significant activation, evidenced by increased phosphorylation of IRE1α, enhanced splicing of XBP1 mRNA, and elevation of the active transcription factor XBP1s protein levels (Fig. 7B-D). Moreover, the expression of the active form of ATF6, specifically the cleaved N-terminal 50 kDa fragment of ATF6 (N-ATF6), was significantly upregulated in response to TiPs (Fig. 7B and D). Upon co-treatment with OST, the PERK signaling cascade was significantly inhibited, as evidenced by reduced phosphorylation of PERK and eIF2α, and decreased expression of ATF4, while the IRE1α and ATF6 signaling did not exhibit marked alterations (Fig. 7A-D).

To further determine the impact of OST on UPR activation in osteoblasts exposed to TiPs, we examined the effects of varying OST concentrations on all three branches following co-treatment with TiPs for 12 h. As shown in Fig. 7E-H, OST treatment inhibited the activation of the PERK signaling cascade in a dose-dependent manner, while it did not significantly affect the IRE1α and ATF6 branches. These findings suggested that OST might primarily exert its effects through the inhibition of PERK signaling activation. To test this conjecture, we conducted molecular docking studies using AutoDock to investigate the potential interaction between OST and PERK. As shown in Fig. 7I, the docking analysis revealed a strong binding affinity of OST towards the PERK kinase domain, with the highest binding energy of -8.1 kcal/mol. Moreover, the docking results indicated that OST interacts with similar binding sites and amino acid residues in the PERK kinase domain as those targeted by the selective PERK inhibitor GSK2606414 (Fig. 7I and J) (Axten et al. 2012). These findings suggested that OST might attenuate TiPs-induced osteogenic impairment by mitigating endoplasmic reticulum stress through the inhibition of PERK signaling activation.

### PERK signaling mediated the protective effects of OST against TiPs-induced osteogenic impairment

**Fig. 8:**
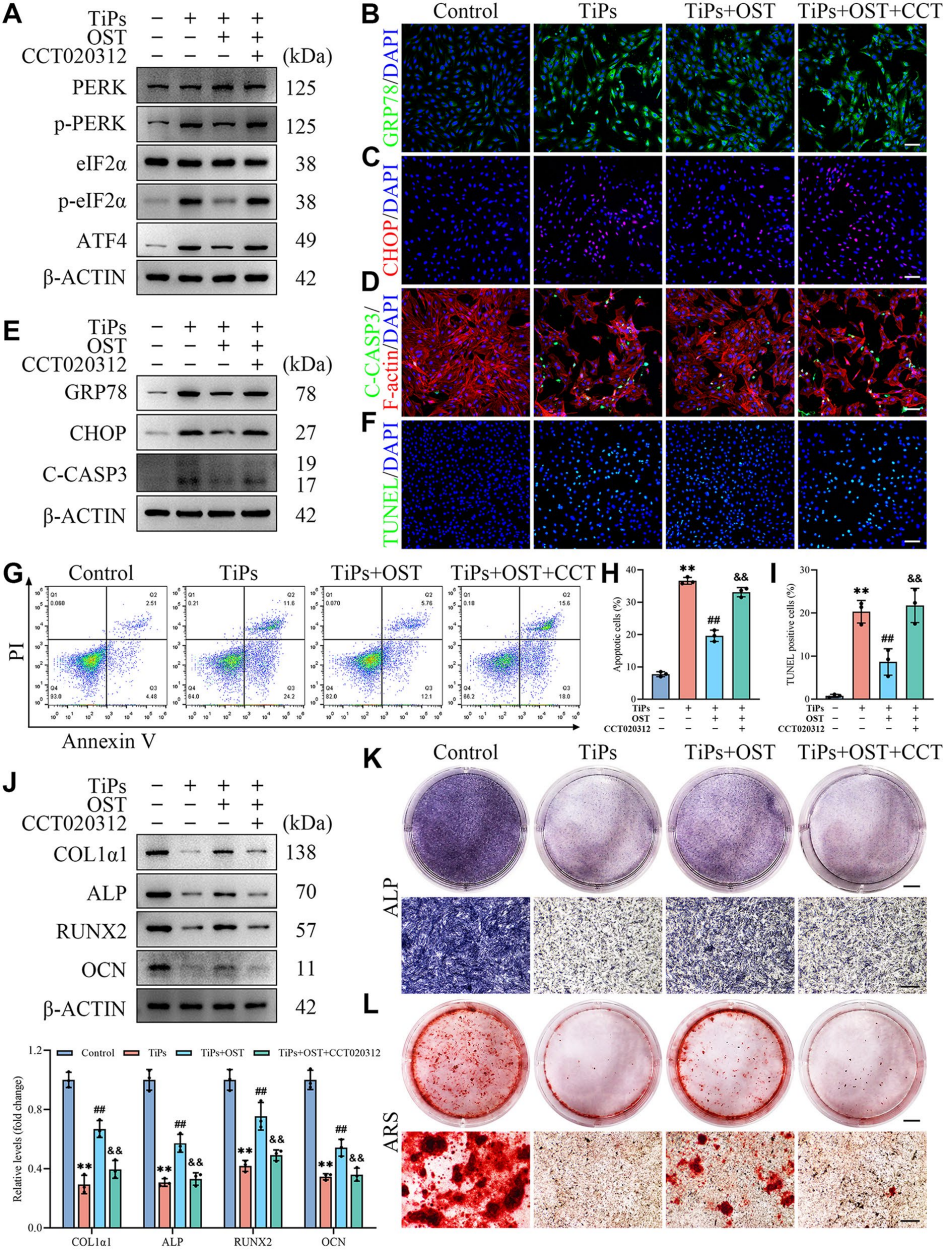
The selective PERK agonist CCT020312 abrogated the protective effects of OST against TiPs-induced osteogenic impairment in vitro. (**A**) Western blots showing the protein levels of PERK, p-PERK, eIF2α, p-eIF2α, and ATF4 in osteoblasts exposed TiPs (50 µg/ml) following treatment with OST (20 µM) or OST combined with CCT020312 (10 µM) for 24 h. (**B**) Representative images of GRP78 immunofluorescence staining (green) in osteoblasts treated as intended. Scale bar, 100 μm. (**C**) Representative images of CHOP immunofluorescence staining (red) in osteoblasts treated as intended. Scale bar, 100 μm. (**D**) Representative images of C-CASP3 immunofluorescence staining (green) in osteoblasts treated as intended, with F-actin labeled in red using phalloidin. Scale bar, 100 μm. (**E**) Western blots showing the protein levels of GRP78, CHOP, and C-CASP3 in osteoblasts treated as intended. (**F**) Representative images of TUNEL staining in osteoblasts treated as intended. Scale bar, 100 μm. (**G-H**) Flow cytometry analysis of apoptosis levels in osteoblasts treated as intended, using Annexin V/PI staining for detection. (**I**) Quantitative analysis of the TUNEL staining results of (F). (**J**) Western blot analysis of the expression of osteogenesis-related proteins (COL1α1, ALP, RUNX2, and OCN) in osteoblasts treated as intended following 3 days of osteogenic induction. (**K**) Representative images of ALP staining of osteoblasts treated as intended following 7 days of osteogenic induction. Scale bar, 5 mm (upper), 500 μm (lower). (**L**) Representative images of ARS staining of osteoblasts treated as intended following 14 days of osteogenic induction. Scale bar, 5 mm (upper), 500 μm (lower). *n* = 3. The selected images reflect typical examples from each group, closely representing the median degree as per statistical analysis. Data are presented as mean ± SD. One-way ANOVA with Tukey’s *post hoc* test. ^**^P < 0.01 *versus* the Control group. ^##^*P* < 0.01 *versus* the TiPs group. ^&&^*P* < 0.01 *versus* the TiPs + OST group

To confirm that the protective effects of OST on stressed osteoblasts upon TiPs exposure are mediated through the inhibition of the PERK signaling cascade, we further conducted additional rescue experiments in vitro using the selective PERK agonist CCT020312 (Bruch et al. 2017). The cell counting kit-8 (CCK-8) results showed that CCT020312 at a concentration of 10 µM effectively counteracted the rescuing effects of OST on osteoblast viability in the presence of TiPs, without exhibiting obvious cytotoxicity (Fig. S5A and S5B). Further Western blot analysis confirmed that CCT020312 effectively abrogated the inhibitory effects of OST on the activation of the PERK signaling cascade triggered by TiPs in osteoblasts (Fig. 8A and S5C). As shown in Fig. 8B-E, S5D, and S5E, OST treatment significantly suppressed the expression of ER stress and apoptosis markers in osteoblasts exposed to TiPs, whereas co-treatment with CCT020312 blunted these alterations in expression. Moreover, TUNEL staining and Flow cytometry analysis further demonstrated that CCT020312 abolished the OST-mediated an-apoptotic effects in osteoblasts subjected to TiPs exposure (Fig. 8F-I).

Next, we investigated the osteogenic functionality of osteoblasts under these conditions. As shown in Fig. 8J, Western blot analysis showed that co-treatment with CCT020312 resulted in a downregulation of the osteogenesis-related protein expression in osteoblasts exposed to TiPs compared to treatment with OST alone. Furthermore, ALP staining and ARS staining revealed that the favorable effects of OST on osteogenesis in TiPs-treated osteoblasts were markedly diminished upon co-treatment with CCT020312 (Fig. 8K and L, S5F, and S5G). To further explore this, we assessed the impact of CCT020312 on the osteogenic effects of OST in the absence of TiPs. As shown in Fig S5H and S5I, ALP staining revealed that the chosen dose of CCT020312 did not adversely impact the osteogenic effects of OST, suggesting that this dose allows for selective modulation of the PERK signaling pathway without compromising the osteogenic potential of OST, further supporting the validity of our findings.

**Fig. 9:**
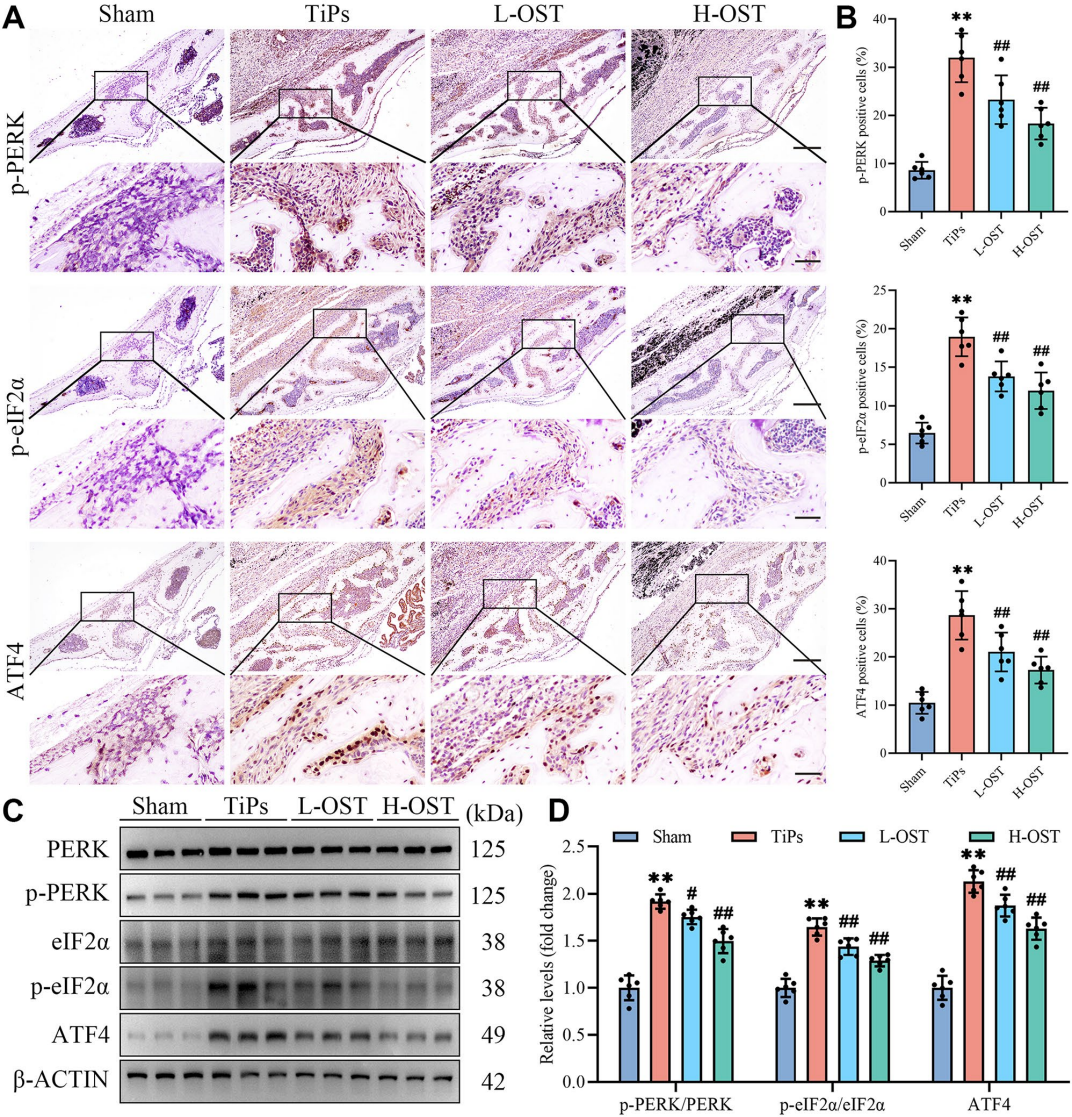
OST treatment repressed TiPs-induced activation of the PERK signaling cascade in mouse calvaria. (**A**) Representative images of IHC staining for p-PERK, p-eIF2α, and ATF4. Scale bar, 200 μm (upper), 50 μm (lower). (**B**) Quantitative analysis of the results of IHC staining for p-PERK, p-eIF2α, and ATF4. (**C-D**) Western blot analysis of the expression of PERK, p-PERK, eIF2α, p-eIF2α, and ATF4 in calvarial bone tissue samples from each group. *n* = 6. The selected images reflect typical examples from each group, closely representing the median degree as per statistical analysis. Data are presented as mean ± SD. One-way ANOVA with Tukey’s *post hoc* test. ^**^P < 0.01 *versus* the Sham group. ^#^*P* < 0.05 and ^##^*P* < 0.01 *versus* the TiPs group

In addition, in vivo IHC staining and Western blot analysis demonstrated that OST treatment inhibited TiPs-induced PERK activation in mouse calvaria, further corroborating our in vitro findings (Fig. 9A-D). Collectively, these results substantiate that PERK signaling plays a crucial role in mediating the protective effects of OST against TiPs-induced osteogenic impairment.

## Discussion

Total joint arthroplasty is recognized as one of the most successful surgical inventions of the 20th century and represents the most effective treatment for end-stage joint diseases (Learmonth et al. 2007). However, aseptic prosthesis loosening remains the most common long-term complication following arthroplasty, serving as a leading cause of late failure and revision surgery. Currently, the only definitive clinical treatment for aseptic loosening is revision arthroplasty (Goodman et al. 2022). Unfortunately, revision arthroplasty is associated with higher surgical complexity, greater cost, and poorer postoperative outcomes compared to primary arthroplasty (Calek et al. 2022). Thus, an in-depth investigation into the pathogenesis of aseptic loosening and the identification of potential therapeutic agents are expected to provide new insights and strategies to address this clinical challenge. The development of aseptic loosening involves a complex biological process, with wear particle-induced osteolysis serving as the central mechanism (Anil et al. 2022; Yu et al. 2024a, b). Wear particles trigger adverse biological responses around the prosthesis, disrupting the balance between bone formation and resorption. While previous studies have predominantly focused on the inflammation response and increased osteoclastic bone resorption caused by wear particles, there has been relatively limited attention on osteoblast-mediated bone formation (Wang et al. 2015a, b, c). It is essential to acknowledge that the detrimental effects of wear particles on osteoblast viability and functionality play a critical role in the development of periprosthetic osteolysis. Our earlier studies have established that ER stress is involved in wear particle-induced osteogenic impairment, underscoring the potential for targeting ER stress in osteoblasts as a promising strategy for preventing and treating aseptic loosening (Wang et al. 2013; Yu et al. 2023a, b, 2024a, b).

Currently, there are no FDA-approved non-surgical pharmacological therapies available for the treatment of aseptic loosening, driving researchers to actively seek potential therapeutic agents for this condition (Goodman et al. 2022). In recent years, natural compounds have garnered increasing attention for their potential to arrest particle-associated periprosthetic osteolysis due to their favorable biosafety and bioactivity (Goodman and Gallo 2019). OST, a natural coumarin derivative, has been reported to possess beneficial osteogenic properties and the ability to modulate ER stress adaption (Xu et al. 2020, 2023, 2024; Chen et al. 2023; Liu et al. 2023). Given this, we conducted this study to evaluate the effects of OST on ER stress-mediated osteogenic impairment in the context of wear particle-indued osteolysis. Our findings revealed that OST treatment ameliorated the impairment of osteogenesis caused by TiPs in both in vivo and in vitro models. Moreover, we observed that OST effectively alleviated ER stress and reduced apoptosis levels in osteoblasts exposed to TiPs while enhancing their osteogenic functionality. Furthermore, we demonstrated that the protective effects of OST against ER stress-mediated osteogenic impairment in osteoblasts upon TiPs exposure are mediated through the PERK signaling cascade. Aligned with our findings, research by Xu and colleagues demonstrated that OST could mitigate porcine circovirus type 2-induced apoptosis by suppressing the ER stress-dependent PERK pathway (Xu et al. 2020, 2023). Interestingly, our results showed that OST treatment did not appear to affect the activation of IRE1α and ATF6 branches of the ER stress response in osteoblasts exposed to TiPs. Furthermore, molecular docking analyses revealed that OST exhibits a high affinity for the PERK kinase domain and shares similar docking sites and binding postures with the selective PERK inhibitor GSK2606414, suggesting that OST may specifically act as a potential inhibitor of the PERK signaling pathway (Axten et al. 2012).

Exposure to nanoparticle exposure is known to induce ER stress, which is considered one of the main factors contributing to nanotoxicity (Cao et al. 2017). Metal wear particles retrieved from periprosthetic tissues of patients with aseptic loosening are typically nanometer-sized, which can be internalized by osteoblasts but cannot be digested by cells, potentially inducing sustained intracellular stress (Doorn et al. 1998). If ER stress persists and the UPR activation fails to restore ER homeostasis, it can trigger pathways leading to ER stress-induced apoptosis (Hetz 2012). During this process, the PERK signaling cascade is regarded as the primary pathway involved in mediating ER stress-mediated apoptosis (Tabas and Ron 2011). Prolonged PERK activation can lead to excessive expression of the downstream effector CHOP, which is the leading mediator of ER stress-induced cell death (Iurlaro and Munoz-Pinedo 2016). CHOP negatively regulates the expression of anti-apoptotic factors such as BCL2, while simultaneously upregulating pro-apoptotic factors such as BAX, leading to a shift in the BCL2/BAX ratio towards apoptosis (Hetz 2012). Moreover, CHOP overexpression impairs osteoblastic function by forming heterodimers with C/EBPβ, thereby inhibiting the DNA-binding activity and RUNX2-binding activity of C/EBPβ (Yu et al. 2023a, b). Our study demonstrated that OST treatment effectively inhibits TiPs-induced PERK activation and downregulates downstream CHOP expression, which likely explains the observed reduction in BAX expression, the increase in BCL2 expression, and the improvement in osteogenic function in osteoblasts. In addition, a study by Lv et al. reported that local administration of OST inhibits tricalcium phosphate particle-induced inflammatory response and osteoclastogenesis in mouse calvaria, potentially through inhibition of the ER stress signaling pathway (Lv et al. 2016). However, whether the PERK signaling cascade is involved in this process remains unclear. Moreover, our previous studies have demonstrated that ER stress also plays a critical role in mediating the inflammation response and osteoclast activation around the prosthesis induced by wear particles, involving the regulation of macrophages, fibroblasts, and osteoclasts (Wang et al. 2013, 2015a, b, c; Liu et al. 2016). Therefore, the effects and regulatory mechanisms of OST on ER stress in other cell types require further investigation to fully understand the therapeutic potential of OST in arresting aseptic loosening.

Conceptually, ER stress can be targeted pharmacologically in two ways: either by directly affecting the accumulation of misfolded proteins within the ER, or by modulating UPR, which are mediated by ER transmembrane sensors such as PERK, IRE1α, and ATF6 (Marciniak et al. 2022). In our previous study, we demonstrated the efficacy of 4-PBA, widely recognized as an ER stress inhibitor, in ameliorating wear particle-induced osteogenic impairment (Yu et al. 2023a, b). 4-PBA is known to function as a chemical chaperone, stabilizing protein conformation and facilitating the transport of misfolded proteins out of the ER for degradation (Cortez and Sim 2014). However, if chaperones are insufficient to fully prevent the accumulation of unfolded proteins, UPR signaling cascades, particularly through PERK, IRE1α, and ATF6, are triggered. While UPR initially serves as a protective mechanism to restore ER homeostasis, prolonged or excessive activation leads to apoptotic cell death (Hetz and Papa 2018). Thus, pharmacological strategies aimed at modulating UPR signaling offering a promising avenue for the treatment of ER stress-related diseases. In this study, we found that OST specifically modulates the PERK signaling within the UPR, offering a more targeted approach to mitigating ER stress. This specificity may reduce the risk of disrupting other essential cellular processes regulated by the UPR, thereby minimizing potential side effects. Moreover, as a naturally occurring compound, OST likely offers a favorable safety profile and superior biocompatibility compared to synthetic drugs. Consequently, OST emerges as a promising candidate and a more attractive option for long-term use in improving outcomes of patients following total joint arthroplasty, especially in conditions associated with aseptic loosening.

Although this study has shown the potential of OST in modulating ER stress in the context of wear particle-induced osteolysis, it is crucial to acknowledge that ER stress inhibition represents only one aspect of its multifaceted therapeutic effects. In previous studies, OST has been shown to regulate osteoblast activity through a variety of mechanisms, such as modulation of Wnt/β-catenin, BMP-2/Smad, cAMP/CREB, estrogen receptor, and ERK1/2-MAPK signaling pathways (Zhang et al. 2017; Tang et al. 2010; Zheng et al. 2024; Kuo et al. 2005; Jia et al. 2016). Our research is pioneering in proposing that OST can regulate osteoblast activity through the modulation of ER stress, adding a novel dimension to our understanding of its therapeutic potential. In addition, OST has also been reported the potential to inhibit the release of inflammatory cytokines and RANKL-induced osteoclastogenesis, facilitate the transition from M1 to M2 macrophage phenotypes, and enhance the immunomodulatory properties of bone marrow-derived stem cells (Wang et al. 2020, 2023; Ma et al. 2019; Yu et al. 2021). Given these findings, further investigations are warranted to explore the potential effects of OST on various cell types in the context of wear particle-induced osteolysis and to fully elucidate its therapeutic potential in aseptic loosening.

This study has several limitations that should be acknowledged. Firstly, as mentioned above, wear particles can interact with various cell types, triggering complex and adverse local responses at both cellular and molecular levels (Goodman and Gallo 2019). While our study focused on the direct effects of OST on osteoblast viability and function, it is important to note that inflammatory responses play a pivotal role in the pathogenesis of periprosthetic osteolysis, particularly in macrophage-driven processes such as osteoclast activation and bone resorption (Goodman et al. 2022; Cong et al. 2023). Given OST’s anti-inflammatory properties, future studies should further investigate its effects on other cell types, especially macrophages, to better understand its broader therapeutic potential in periprosthetic osteolysis (Zafar et al. 2021). Secondly, the absence of a positive-control group in our research represents a notable limitation. However, it is necessary to emphasize that the lack of FDA-approved pharmacological therapies for arresting particle-associated periprosthetic osteolysis remains a substantial challenge in this field (Goodman et al. 2022). Thirdly, different types of prosthetic materials generate wear particles with distinct characteristics (Jiang et al. 2013). This study focused on metal wear particles and did not extensively explore other types of particles, such as polyethylene and ceramic particles. Future investigations should incorporate diverse wear particle types to validate and extend our findings comprehensively.

## Conclusion

**Fig. 10:**
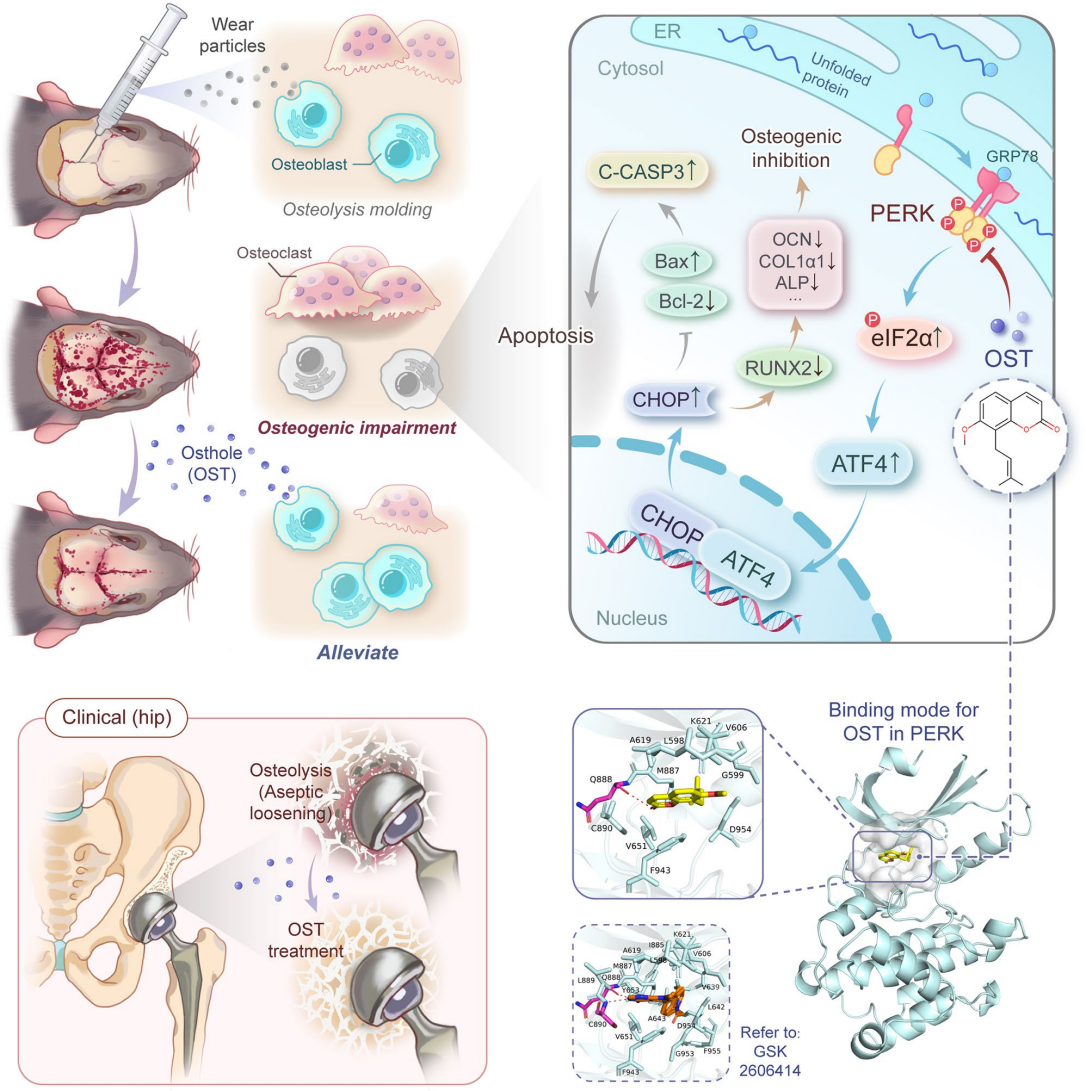
Schematic diagram illustrating the protective effects of OST against wear particle-induced osteogenic impairment via inhibition of the ER stress-dependent PERK signaling cascade

In the present study, we observed that OST treatment could effectively mitigate TiPs-induced osteolysis in a mouse calvarial model. Furthermore, we found that OST treatment ameliorates ER stress-mediated apoptosis and osteogenesis inhibition in osteoblasts exposed to TiPs in vitro and in vivo. Mechanistically, we demonstrated that OST exerts bone-sparing effects on stressed osteoblasts upon TiPs exposure by specifically suppressing the ER stress-dependent PERK signaling cascade (Fig. 10). These findings suggested that OST might be a potential therapeutic agent for addressing wear particle-induced osteogenic impairment, providing a novel alternative strategy for managing aseptic prosthesis loosening.

## Electronic supplementary material

Below is the link to the electronic supplementary material.

Supplementary Material 1

## Data Availability

No datasets were generated or analysed during the current study.

## Abbreviations

α-MEM: Alpha-Minimal Essential Medium
ANOVA: Analysis of variance
ALP: Alkaline phosphatase
ALT: Alanine aminotransferase
ARS: Alizarin red S
AST: Aspartate aminotransferase
ATF4: Activating transcription factor 4
ATF6: Activating transcription factor 6
BMD: Bone mineral density
BP: Biological process
BUN: Blood urea nitrogen
BV/TV: Bone volume-to-total volume
CC: Cellular component
CC50: Half-maximal cytotoxic concentration
C-CASP3: Cleaved-caspase 3
CCK-8: Cell counting kit-8
CHOP: C/EBP-homologous protein
COL1α1: Collagen type I alpha 1
DAPI: 4,6-Diamidino-2-phenylindole
DMSO: Dimethyl sulfoxide
EDS: Energy dispersive spectrometry
eIF2α: Eukaryotic translation initiation factor 2 alpha
DEGs: Differentially expressed genes
EBS/BS: Eroded bone surface per bone surface
ER: Endoplasmic reticulum
FBS: Fetal bovine serum
FDA: Food and Drug Administration
FPKM: Fragments Per Kilobase of transcript per Million mapped reads
GO: Gene ontology
GRP78: Glucose-regulated protein 78 kDa
GSEA: Gene set enrichment analysis
H&E: Bematoxylin & eosin
IC50: Half-maximal inhibitory concentration
IHC: Immunohistochemistry
IRE1α: Inositol-requiring enzyme 1 alpha
KEGG: Kyoto encyclopedia of genes and genomes
MAR: Mineral apposition rate
MF: Molecular function
N-ATF6: N-terminal 50 kDa fragment of ATF6
OCN: Osteocalcin
Oc.S/BS: Osteoclast surface per bone surface
OCT: Optimum cutting temperature
OST: Osthole
PBS: Phosphate-buffered saline
PCA: Principal component analysis
PCR: Polymerase chain reaction
PERK: Protein kinase R-like ER kinase
PFA: Paraformaldehyde
PI: Propidium iodide
RNA-seq: RNA sequencing
ROI: Region of interest
RUNX2: Runt-related transcription factor 2
SCr: Serum creatine
SEM: Scanning electron microscopy
SD: Standard deviation
TEM: Transmission electron microscopy
TRAP: Tartrate-resistant acid phosphatase
TUNEL: TdT-mediated dUTP nick end-labeling
TiPs: TiAl_6_V_4_ particles
UPR: Unfolded protein response
XBP1s: The spliced form of X box-binding protein 1
XBP1u: The unspliced form of X box-binding protein 1

## References

[CR48] An Y, Xu C, Liu W, Jiang J, Ye P, Yang M, Zhu W, Yu J, Yu M, Sun W, Hong J, Qiu H, Wei W, Zhang S. Angiotensin II type-2 receptor attenuates liver fibrosis progression by suppressing IRE1alpha-XBP1 pathway. Cell Signal. 2024;113:110935. 37866666 10.1016/j.cellsig.2023.110935

[CR2] Anil U, Singh V, Schwarzkopf R. Diagnosis and detection of subtle aseptic loosening in total hip arthroplasty. J Arthroplasty. 2022;37(8):1494–500. 35189292 10.1016/j.arth.2022.02.060

[CR49] Axten JM, Medina JR, Feng Y, Shu A, Romeril SP, Grant SW, Li WH, Heerding DA, Minthorn E, Mencken T, Atkins C, Liu Q, Rabindran S, Kumar R, Hong X, Goetz A, Stanley T, Taylor JD, Sigethy SD, Tomberlin GH, Hassell AM, Kahler KM, Shewchuk LM, Gampe RT. Discovery of 7-methyl-5-(1-[3-(trifluoromethyl)phenyl]acetyl-2,3-dihydro-1H-indol-5-yl)-7H-pyrrolo[2,3-d]pyrimidin-4-amine (GSK2606414), a potent and selective first-in-class inhibitor of protein kinase R (PKR)-like endoplasmic reticulum kinase (PERK). J Med Chem. 2012;55(16):7193–207. 22827572 10.1021/jm300713s

[CR53] Bruch J, Xu H, Rosler TW, De Andrade A, Kuhn PH, Lichtenthaler SF, Arzberger T, Winklhofer KF, Muller U, Hoglinger GU. PERK activation mitigates tau pathology in vitro and in vivo. EMBO Mol Med. 2017;9(3):371–84. 28148553 10.15252/emmm.201606664 PMC5331260

[CR55] Calek AK, Schofl T, Zdravkovic V, Zurmuhle P, Ladurner A. Aseptic revision of total hip arthroplasty with a single modular femoral stem and a modified extended Trochanteric Osteotomy-Treatment Assessment with the Forgotten Joint Score-12. Arthroplast Today. 2022;15:159–66. 35601994 10.1016/j.artd.2022.03.024 PMC9121271

[CR57] Cao Y, Long J, Liu L, He T, Jiang L, Zhao C, Li Z. A review of endoplasmic reticulum (ER) stress and nanoparticle (NP) exposure. Life Sci. 2017;186:33–42. 28782531 10.1016/j.lfs.2017.08.003

[CR1] Carender CN, Bothun CE, Sierra RJ, Trousdale RT, Abdel MP, Bedard NA. Contemporary aseptic revision total hip arthroplasty in patients =50 years of age: results of 500 cases. J Bone Joint Surg Am. 2024;106(12):1108–16. 38687829 10.2106/JBJS.23.01467

[CR19] Chen J, Liao X, Gan J. Review on the protective activity of osthole against the pathogenesis of osteoporosis. Front Pharmacol. 2023;14:1236893. 37680712 10.3389/fphar.2023.1236893 PMC10481961

[CR51] Chen Y, Li X, Yang M, Liu SB. Research progress on morphology and mechanism of programmed cell death. Cell Death Dis. 2024;15(5):327. 38729953 10.1038/s41419-024-06712-8 PMC11087523

[CR71] Cong Y, Wang Y, Yuan T, Zhang Z, Ge J, Meng Q, Li Z, Sun S. Macrophages in aseptic loosening: characteristics, functions, and mechanisms. Front Immunol. 2023;14:1122057. 36969165 10.3389/fimmu.2023.1122057 PMC10030580

[CR63] Cortez L, Sim V. The therapeutic potential of chemical chaperones in protein folding diseases. Prion. 2014;8(2):197–202. 24818993 10.4161/pri.28938 PMC4189890

[CR33] Deng Z, Wang S, Li M, Fu G, Liu C, Li S, Jin J, Lyu FJ, Ma Y, Zheng Q. A Modified Murine Calvarial Osteolysis Model Exposed to Ti Particles in Aseptic Loosening, Biomed Res Int 2020 (2020) 3403489. 10.1155/2020/3403489 PMC7468620 32908884

[CR34] Doorn PF, Campbell PA, Worrall J, Benya PD, McKellop HA, Amstutz HC. Metal wear particle characterization from metal on metal total hip replacements: transmission electron microscopy study of periprosthetic tissues and isolated particles. J Biomed Mater Res. 1998;42(1):103–11. 9740012 10.1002/(sici)1097-4636(199810)42:1<103::aid-jbm13>3.0.co;2-m

[CR27] Gao LN, An Y, Lei M, Li B, Yang H, Lu H, Chen FM, Jin Y. The effect of the coumarin-like derivative osthole on the osteogenic properties of human periodontal ligament and jaw bone marrow mesenchymal stem cell sheets. Biomaterials. 2013;34(38):9937–51. 24095254 10.1016/j.biomaterials.2013.09.017

[CR56] Goodman SB, Gallo J. Periprosthetic Osteolysis: Mechanisms, Prevention and Treatment, J Clin Med 8(12) (2019) 2091. 10.3390/jcm8122091 PMC6947309 31805704

[CR14] Goodman SB, Gibon E, Gallo J, Takagi M. Macrophage polarization and the osteoimmunology of Periprosthetic Osteolysis. Curr Osteoporos Rep. 2022;20(1):43–52. 35133558 10.1007/s11914-022-00720-3

[CR7] Hetz C. The unfolded protein response: controlling cell fate decisions under ER stress and beyond. Nat Rev Mol Cell Biol. 2012;13(2):89–102. 22251901 10.1038/nrm3270

[CR8] Hetz C, Papa FR. The unfolded protein response and cell Fate Control. Mol Cell. 2018;69(2):169–81. 29107536 10.1016/j.molcel.2017.06.017

[CR58] Iurlaro R, Munoz-Pinedo C. Cell death induced by endoplasmic reticulum stress. FEBS J. 2016;283(14):2640–52. 26587781 10.1111/febs.13598

[CR66] Jia M, Li Y, Xin HL, Hou TT, Zhang ND, Xu HT, Zhang QY, Qin LP. Estrogenic activity of osthole and imperatorin in MCF-7 cells and their osteoblastic effects in Saos-2 cells. Chin J Nat Med. 2016;14(6):413–20. 27473958 10.1016/S1875-5364(16)30037-1

[CR72] Jiang Y, Jia T, Gong W, Wooley PH, Yang SY. Effects of Ti, PMMA, UHMWPE, and co-cr wear particles on differentiation and functions of bone marrow stromal cells. J Biomed Mater Res A. 2013;101(10):2817–25. 24039045 10.1002/jbm.a.34595 PMC3775288

[CR32] Jiang H, Wang Y, Deng Z, Jin J, Meng J, Chen S, Wang J, Qiu Y, Guo T, Zhao J. Construction and evaluation of a murine calvarial osteolysis model by exposure to CoCrMo particles in aseptic loosening. J Vis Exp (132) (2018). 10.3791/56276 PMC5931316 29553545

[CR23] Jin ZX, Liao XY, Da WW, Zhao YJ, Li XF, Tang DZ. Osthole enhances the bone mass of senile osteoporosis and stimulates the expression of osteoprotegerin by activating beta-catenin signaling. Stem Cell Res Ther. 2021;12(1):154. 33640026 10.1186/s13287-021-02228-6 PMC7912492

[CR36] Jin Z, Da W, Zhao Y, Wang T, Xu H, Shu B, Gao X, Shi Q, Ma Y, Zhang Y, Wang Y, Tang D. Role of skeletal muscle satellite cells in the repair of osteoporotic fractures mediated by beta-catenin. J Cachexia Sarcopenia Muscle. 2022;13(2):1403–17. 35178895 10.1002/jcsm.12938 PMC8977954

[CR18] Kong L, Li XT, Ni YN, Xiao HH, Yao YJ, Wang YY, Ju RJ, Li HY, Liu JJ, Fu M, Wu YT, Yang JX, Cheng L. Transferrin-modified Osthole PEGylated liposomes Travel the blood-brain barrier and mitigate Alzheimer’s Disease-Related Pathology in APP/PS-1 mice. Int J Nanomed. 2020;15:2841–58. 10.2147/IJN.S239608 PMC7186891 32425521

[CR65] Kuo PL, Hsu YL, Chang CH, Chang JK. Osthole-mediated cell differentiation through bone morphogenetic protein-2/p38 and extracellular signal-regulated kinase 1/2 pathway in human osteoblast cells. J Pharmacol Exp Ther. 2005;314(3):1290–9. 15956019 10.1124/jpet.105.085092

[CR54] Learmonth ID, Young C, Rorabeck C. The operation of the century: total hip replacement. Lancet. 2007;370(9597):1508–19. 17964352 10.1016/S0140-6736(07)60457-7

[CR61] Liu G, Liu N, Xu Y, Ti Y, Chen J, Chen J, Zhang J, Zhao J. Endoplasmic reticulum stress-mediated inflammatory signaling pathways within the osteolytic periosteum and interface membrane in particle-induced osteolysis. Cell Tissue Res. 2016;363(2):427–47. 26004143 10.1007/s00441-015-2205-9 PMC4735257

[CR28] Liu J, Wu Q, Wu Q, Zhong G, Liang Y, Gu Y, Hu Y, Wang W, Hao N, Fang S, Li W, Pan H, Wang Q, Fang J. Modulating endoplasmic reticulum stress in APP/PS1 mice by Gomisin B and Osthole in Bushen-Yizhi formula: synergistic effects and therapeutic implications for Alzheimer’s disease. Phytomedicine. 2023;119:155023. 37586159 10.1016/j.phymed.2023.155023

[CR31] Lv S, Zhang Y, Yan M, Mao H, Pan C, Gan M, Fan J, Wang G. Inhibition of osteolysis after local administration of osthole in a TCP particles-induced osteolysis model. Int Orthop. 2016;40(7):1545–52. 26498175 10.1007/s00264-015-3021-2

[CR69] Ma Y, Wang L, Zheng S, Xu J, Pan Y, Tu P, Sun J, Guo Y. Osthole inhibits osteoclasts formation and bone resorption by regulating NF-kappaB signaling and NFATc1 activations stimulated by RANKL. J Cell Biochem. 2019;120(9):16052–61. 31081953 10.1002/jcb.28886

[CR62] Marciniak SJ, Chambers JE, Ron D. Pharmacological targeting of endoplasmic reticulum stress in disease. Nat Rev Drug Discov. 2022;21(2):115–40. 34702991 10.1038/s41573-021-00320-3

[CR35] Mediero A, Frenkel SR, Wilder T, He W, Mazumder A, Cronstein BN. Adenosine A2A receptor activation prevents wear particle-induced osteolysis. Sci Transl Med. 2012;4(135):135ra165. 10.1126/scitranslmed.3003393 PMC3386559 22623741

[CR52] Ndozangue-Touriguine O, Hamelin J, Breard J. Cytoskeleton and apoptosis. Biochem Pharmacol. 2008;76(1):11–8. 18462707 10.1016/j.bcp.2008.03.016

[CR3] O’Neill SC, Queally JM, Devitt BM, Doran PP. O’Byrne, the role of osteoblasts in peri-prosthetic osteolysis. Bone Joint J. 2013;95–B(8):1022–6. 23908414 10.1302/0301-620X.95B8.31229

[CR43] Pflanz D, Birkhold AI, Albiol L, Thiele T, Julien C, Seliger A, Thomson E, Kramer I, Kneissel M, Duda GN, Kornak U, Checa S, Willie BM. Sost deficiency led to a greater cortical bone formation response to mechanical loading and altered gene expression. Sci Rep. 2017;7(1):9435. 28842678 10.1038/s41598-017-09653-9 PMC5572735

[CR39] Sato T, Pajarinen J, Lin TH, Tamaki Y, Loi F, Egashira K, Yao Z, Goodman SB. NF-kappaB decoy oligodeoxynucleotide inhibits wear particle-induced inflammation in a murine calvarial model. J Biomed Mater Res A. 2015;103(12):3872–8. 26123702 10.1002/jbm.a.35532 PMC4817851

[CR5] Schwarz DS, Blower MD. The endoplasmic reticulum: structure, function and response to cellular signaling. Cell Mol Life Sci. 2016;73(1):79–94. 26433683 10.1007/s00018-015-2052-6 PMC4700099

[CR21] Singh L, Bhatti R. Signaling pathways involved in the neuroprotective effect of Osthole: evidence and mechanisms. Mol Neurobiol. 2024;61(2):1100–18. 37682453 10.1007/s12035-023-03580-9

[CR9] Szegezdi E, Logue SE, Gorman AM, Samali A. Mediators of endoplasmic reticulum stress-induced apoptosis. EMBO Rep. 2006;7(9):880–5. 16953201 10.1038/sj.embor.7400779 PMC1559676

[CR10] Tabas I, Ron D. Integrating the mechanisms of apoptosis induced by endoplasmic reticulum stress. Nat Cell Biol. 2011;13(3):184–90. 21364565 10.1038/ncb0311-184 PMC3107571

[CR26] Tang DZ, Hou W, Zhou Q, Zhang M, Holz J, Sheu TJ, Li TF, Cheng SD, Shi Q, Harris SE, Chen D, Wang YJ. Osthole stimulates osteoblast differentiation and bone formation by activation of beta-catenin-BMP signaling. J Bone Min Res. 2010;25(6):1234–45. 10.1002/jbmr.21 PMC3153131 20200936

[CR44] Tanjaya J, Lord EL, Wang C, Zhang Y, Kim JK, Nguyen A, Baik L, Pan HC, Chen E, Kwak JH, Zhang X, Wu B, Soo C, Ting K. The effects of systemic therapy of PEGylated NEL-Like protein 1 (NELL-1) on Fracture Healing in mice. Am J Pathol. 2018;188(3):715–27. 29294300 10.1016/j.ajpath.2017.11.018 PMC5840496

[CR47] Taylor SE, Shah M, Orriss IR. Generation of rodent and human osteoblasts. Bonekey Rep. 2014;3:585. 25396049 10.1038/bonekey.2014.80 PMC4230189

[CR16] Tu Y, Yang Y, Li Y, He C. Naturally occurring coumestans from plants, their biological activities and therapeutic effects on human diseases. Pharmacol Res. 2021;169:105615. 33872808 10.1016/j.phrs.2021.105615

[CR6] Wang M, Kaufman RJ. Protein misfolding in the endoplasmic reticulum as a conduit to human disease. Nature. 2016;529(7586):326–35. 26791723 10.1038/nature17041

[CR11] Wang R, Wang Z, Ma Y, Liu G, Shi H, Chen J, Dong L, Zhao J, Zhang J. Particle-induced osteolysis mediated by endoplasmic reticulum stress in prosthesis loosening. Biomaterials. 2013;34(11):2611–23. 23347837 10.1016/j.biomaterials.2013.01.025

[CR45] Wang Z, Liu N, Liu K, Zhou G, Gan J, Wang Z, Shi T, He W, Wang L, Guo T, Bao N, Wang R, Huang Z, Chen J, Dong L, Zhao J, Zhang J. Autophagy mediated CoCrMo particle-induced peri-implant osteolysis by promoting osteoblast apoptosis. Autophagy. 2015a;11(12):2358–69. 26566231 10.1080/15548627.2015.1106779 PMC4835204

[CR59] Wang Z, Huang Z, Gan J, Liu N, Zhou G, Shi T, Wang Z, Wang R, Bao N, Guo T, Chen J, Zhang J, Dong L, Zhao J. The fibroblast expression of RANKL in CoCrMo-particle-induced osteolysis is mediated by ER stress and XBP1s. Acta Biomater. 2015b;24:352–60. 26112372 10.1016/j.actbio.2015.06.024

[CR60] Wang Z, Liu N, Shi T, Zhou G, Wang Z, Gan J, Guo T, Qian H, Bao N, Zhao J. ER stress mediates TiAl6V4 Particle-Induced Peri-implant Osteolysis by promoting RANKL expression in fibroblasts. PLoS ONE. 2015c;10(9):e0137774. 26366858 10.1371/journal.pone.0137774 PMC4569331

[CR24] Wang P, Ying J, Luo C, Jin X, Zhang S, Xu T, Zhang L, Mi M, Chen D, Tong P, Jin H. Osthole promotes bone fracture healing through activation of BMP signaling in Chondrocytes. Int J Biol Sci. 2017a;13(8):996–1007. 28924381 10.7150/ijbs.19986 PMC5599905

[CR46] Wang Z, Deng Z, Gan J, Zhou G, Shi T, Wang Z, Huang Z, Qian H, Bao N, Guo T, Chen J, Zhang J, Liu F, Dong L, Zhao J. TiAl(6)V(4) particles promote osteoclast formation via autophagy-mediated downregulation of interferon-beta in osteocytes. Acta Biomater. 2017b;48:489–98. 27838463 10.1016/j.actbio.2016.11.020

[CR68] Wang L, Zheng S, Huang G, Sun J, Pan Y, Si Y, Tu P, Xu G, Ma Y, Guo Y. Osthole-loaded N-octyl-O-sulfonyl chitosan micelles (NSC-OST) inhibits RANKL-induced osteoclastogenesis and prevents ovariectomy-induced bone loss in rats. J Cell Mol Med. 2020;24(7):4105–17. 32126148 10.1111/jcmm.15064 PMC7171421

[CR67] Wang F, Yang P, Wan T, Liu C, Zhu Y, Chen X, Liu H. Osthole inhibits M1 Macrophage polarization and attenuates Osteolysis in a mouse Skull Model. Oxid Med Cell Longev. 2023;2023:2975193. 36686380 10.1155/2023/2975193 PMC9851800

[CR42] Wedemeyer C, Xu J, Neuerburg C, Landgraeber S, Malyar NM, von Knoch F, Gosheger G, von Knoch M, Loer F, Saxler G. Particle-induced osteolysis in three-dimensional micro-computed tomography. Calcif Tissue Int. 2007;81(5):394–402. 17952672 10.1007/s00223-007-9077-2

[CR25] Wu B, Zhu XF, Yang XQ, Wang WY, Lu JH. Effects of osthole on osteoporotic rats: a systematic review and meta-analysis. Pharm Biol. 2022;60(1):1625–34. 35980123 10.1080/13880209.2022.2110267 PMC9397480

[CR38] Wu Q, Chen B, Yu X, Wang Z, Sun Z, Duan J, Ding H, Wu W, Bao N, Zhao J. Bone and soft tissue reaction to Co(II)/Cr(III) ions stimulation in a murine calvaria model: a pioneering in vivo study. Acta Biomater. 2023;164:659–70. 37003495 10.1016/j.actbio.2023.03.037

[CR40] Xie J, Hu Y, Li H, Wang Y, Fan X, Lu W, Liao R, Wang H, Cheng Y, Yang Y, Wang J, Liang S, Ma T, Su W. Targeted therapy for peri-prosthetic osteolysis using macrophage membrane-encapsulated human urine-derived stem cell extracellular vesicles. Acta Biomater. 2023;160:297–310. 36773884 10.1016/j.actbio.2023.02.003

[CR29] Xu Y, Sun P, Wan S, Guo J, Zheng X, Sun Y, Fan K, Yin W, Sun N, Li H. The combined usage of Matrine and Osthole inhibited endoplasmic reticulum apoptosis induced by PCV2. BMC Microbiol. 2020;20(1):303. 33046006 10.1186/s12866-020-01986-2 PMC7549248

[CR30] Xu Y, Wan S, Sun P, Khan A, Guo J, Zheng X, Sun Y, Fan K, Yin W, Li H, Sun N. Matrine combined with Osthole inhibited the PERK apoptosis of splenic lymphocytes in PCV2-infected mice model. BMC Vet Res. 2023;19(1):26. 36717886 10.1186/s12917-023-03581-9 PMC9885934

[CR17] Xu T, Yin J, Dai X, Liu T, Shi H, Zhang Y, Wang S, Yue G, Zhang Y, Zhao D, Gao S, Prentki M, Wang L, Zhang D. Cnidii Fructus: a traditional Chinese medicine herb and source of antiosteoporotic drugs. Phytomedicine. 2024;128:155375. 38507853 10.1016/j.phymed.2024.155375

[CR50] Yang F, Tang J, Dai K, Huang Y. Metallic wear debris collected from patients induces apoptosis in rat primary osteoblasts via reactive oxygen species–mediated mitochondrial dysfunction and endoplasmic reticulum stress. Mol Med Rep. 2019;19(3):1629–37. 30628694 10.3892/mmr.2019.9825 PMC6390047

[CR70] Yu Y, Chen M, Yang S, Shao B, Chen L, Dou L, Gao J, Yang D. Osthole enhances the immunosuppressive effects of bone marrow-derived mesenchymal stem cells by promoting the Fas/FasL system. J Cell Mol Med. 2021;25(10):4835–45. 33749126 10.1111/jcmm.16459 PMC8107110

[CR12] Yu X, Ding H, Wang D, Ren Z, Chen B, Wu Q, Yuan T, Liu Y, Zhang L, Zhao J, Sun Z. Particle-induced osteolysis is mediated by endoplasmic reticulum stress-associated osteoblast apoptosis. Chem Biol Interact. 2023a;383:110686. 37659624 10.1016/j.cbi.2023.110686

[CR37] Yu X, Yang B, Chen B, Wu Q, Ren Z, Wang D, Yuan T, Ding H, Ding C, Liu Y, Zhang L, Sun Z, Zhao J. Inhibitory effects of Formononetin on CoCrMo particle-induced osteoclast activation and bone loss through downregulating NF-kappaB and MAPK signaling. Cell Signal. 2023b;106:110651. 36894124 10.1016/j.cellsig.2023.110651

[CR13] Yu X, Ren Z, Wang Y, Yuan G, Hu J, Song L, Pan C, Feng K, Liu Y, Shao L, Zhang L, Wang J, Zhao J, Bao N, Sun Z. Kaempferol attenuates particle-induced osteogenic impairment by regulating ER stress via the IRE1alpha-XBP1s pathway. J Biol Chem. 2024a;300(6):107394. 38768813 10.1016/j.jbc.2024.107394 PMC11223082

[CR15] Yu X, Wu Q, Ren Z, Chen B, Wang D, Yuan T, Ding H, Wang Y, Yuan G, Wang Y, Zhang L, Zhao J, Sun Z. Kaempferol attenuates wear particle-induced inflammatory osteolysis via JNK and p38-MAPK signaling pathways. J Ethnopharmacol. 2024b;318(Pt B):117019. 10.1016/j.jep.2023.117019 10.1016/j.jep.2023.117019 37574017

[CR20] Zafar S, Sarfraz I, Rasul A, Shah MA, Hussain G, Zahoor MK, Shafiq N, Riaz A, Selamoglu Z, Sarker SD. Osthole: a multifunctional natural compound with potential anticancer, antioxidant and anti-inflammatory activities. Mini Rev Med Chem. 2021;21(18):2747–63. 32646359 10.2174/1389557520666200709175948

[CR22] Zhang ZR, Leung WN, Li G, Kong SK, Lu X, Wong YM, Chan CW. Osthole enhances osteogenesis in Osteoblasts by Elevating Transcription Factor Osterix via cAMP/CREB signaling in Vitro and in vivo. Nutrients 9(6) (2017). 10.3390/nu9060588 PMC5490567 28629115

[CR4] Zhang L, Haddouti EM, Welle K, Burger C, Wirtz DC, Schildberg FA, Kabir K. The effects of Biomaterial Implant wear debris on osteoblasts. Front Cell Dev Biol. 2020;8:352. 32582688 10.3389/fcell.2020.00352 PMC7283386

[CR64] Zheng S, Hu G, Zheng J, Li Y, Li J. Osthole accelerates osteoporotic fracture healing by inducing the osteogenesis-angiogenesis coupling of BMSCs via the Wnt/beta-catenin pathway. Phytother Res. 2024;38(8):4022–35. 38873735 10.1002/ptr.8267

[CR41] Zhu L, Wang Z, Sun X, Yu J, Li T, Zhao H, Ji Y, Peng B, Du M. STAT3/Mitophagy Axis coordinates macrophage NLRP3 inflammasome activation and inflammatory bone loss. J Bone Min Res. 2023;38(2):335–53. 10.1002/jbmr.4756 36502520

