# Supplementary material for: Osthole ameliorates wear particle-induced osteogenic impairment by mitigating endoplasmic reticulum stress via PERK signaling cascade
Source: Mol Med. 2024 Dec 20;30:266. doi: 10.1186/s10020-024-01034-z (PMC11660672; doi:10.1186/s10020-024-01034-z)

**Osthole ameliorates wear particle-induced osteogenic impairment by mitigating endoplasmic reticulum stress via PERK signaling cascade**

Xin Yu ^a, 1^, Juan Jiang ^b, 1^, Cheng Li ^a, 1^, Yang Wang ^a, 1^, Zhengrong Ren ^b^, Jianlun Hu ^c^, Tao Yuan ^a^, Yongjie Wu ^b^, Dongsheng Wang ^d^, Ziying Sun ^a^, Qi Wu ^e^, Bin Chen ^f^, Peng Fang ^a^, Hao Ding ^a^, Jia Meng ^a, *^, Hui Jiang ^a, *^, Jianning Zhao ^a, g, *^, Nirong Bao ^a, *^

^a^ Department of Orthopedics, Nanjing Jinling Hospital, Affiliated Hospital of Medical School, Nanjing University, Nanjing, China.

^b^ State Key Laboratory of Pharmaceutical Biotechnology, School of Life Sciences, Nanjing University, Nanjing, China.

^c^ State Key Laboratory of Pharmaceutical Biotechnology, Jiangsu Key Laboratory of Molecular Medicine, Medical School, Nanjing University, Nanjing, China.

^d^ Department of Spine Surgery, Center of Orthopedics, Daping Hospital, Army Medical University (Third Military Medical University), Chongqing, China.

^e^ Department of Vascular Surgery, Beijing Tsinghua Changgung Hospital, School of Clinical Medicine, Tsinghua University, Beijing, China.

^f^ Berlin Institute of Health at Charité - Universitätsmedizin Berlin, Julius Wolff Institute, Berlin, Germany.

^g^ Department of Orthopedics, Nanjing Hospital of Chinese Medicine Affiliated to Nanjing University of Chinese Medicine, Nanjing, China.

^1^ These authors contributed equally to this work.

^*^ Corresponding author.

E-mail:

[michaelmengjia@163.com](mailto:michaelmengjia@163.com) (Jia Meng);

[jianghui16663@163.com](mailto:jianghui16663@163.com) (Hui Jiang);

zhaojianning.0207@163.com (Jianning Zhao);

bnrbnr@sina.com (Nirong Bao).

**Supplementary figure legends**

**
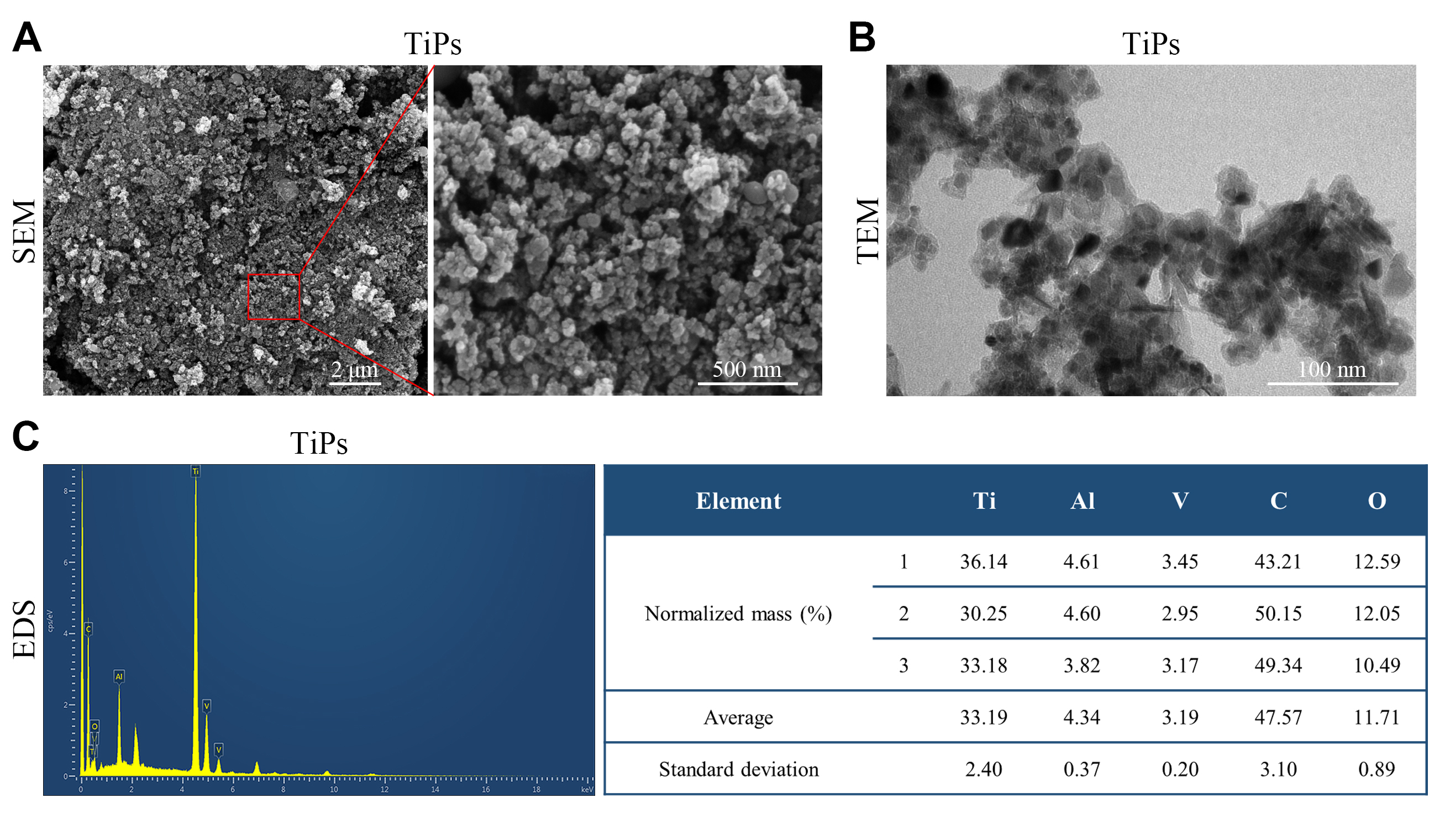
**

**Fig. S1. The physicochemical properties of TiAl_6_V_4_ particles (TiPs).** (**A**) The scanning electron microscopy (SEM) image of TiPs. (**B**) The transmission electron microscopy (TEM) image of TiPs. (**C**) The energy dispersive spectrometry (EDS) and elemental analysis of TiPs.


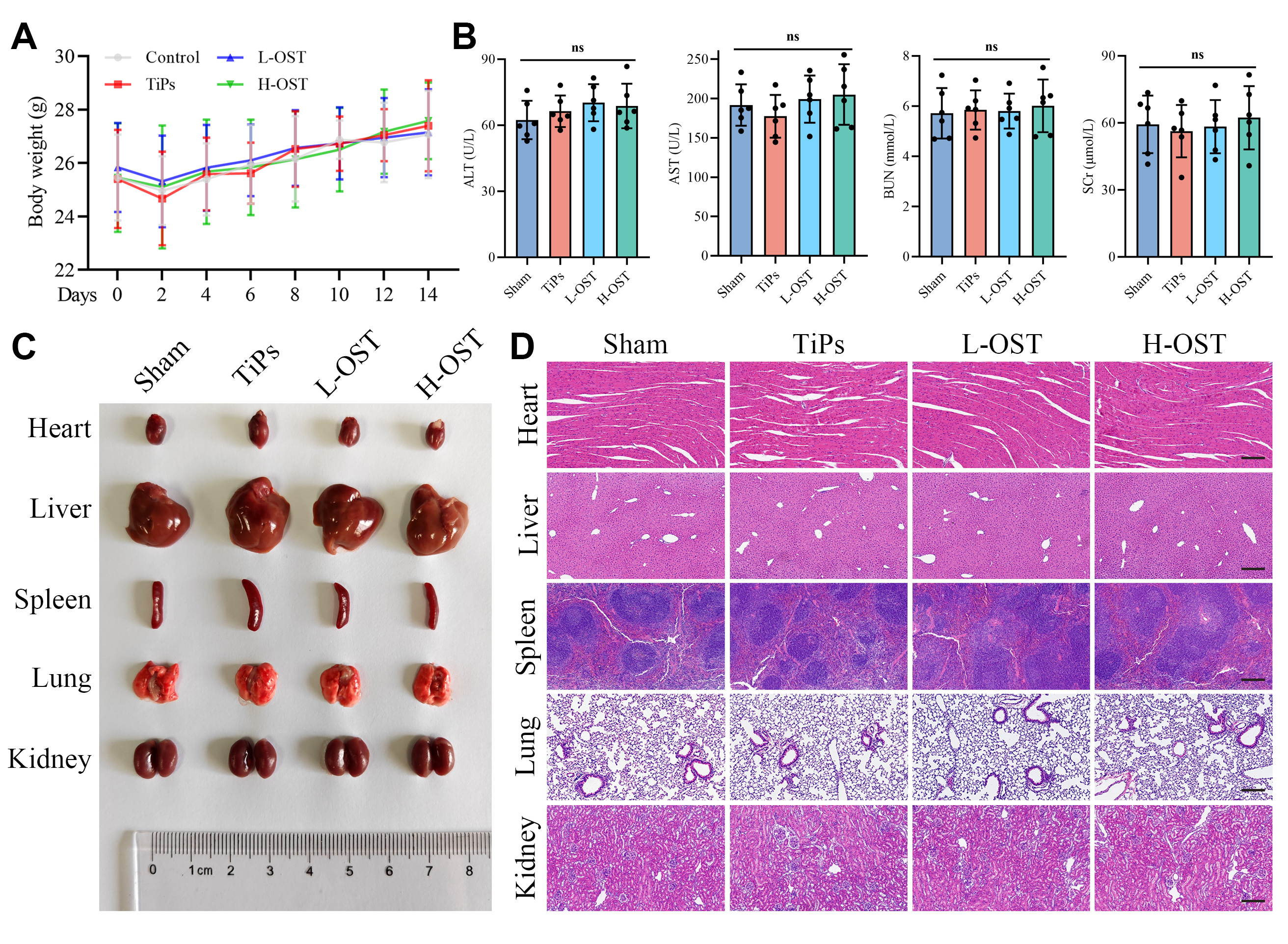


**Fig. S2. Biosafety assessment of OST treatment *in vivo*.** (**A**) Changes in the body weight of mice across different groups over a 14-day treatment period. (**B**) Serum biochemical analysis of alanine aminotransferase (ALT), aspartate aminotransferase (AST), blood urea nitrogen (BUN), and serum creatine (SCr) in mice from different groups following 14 days of treatment. (**C**) Gross view of major organs from mice in different groups, including heart, liver, spleen, lung, and kidney. (**D**) Representative H&E staining images of major organ sections (heart, liver, spleen, lung, and kidney) in different groups. Scale bar, 200 μm. *n* = 6. Data are presented as mean ± SD. Data are presented as mean ± SD. One-way ANOVA with Tukey’s *post hoc* test. *ns*, not statistically significant *versus* the Sham group.


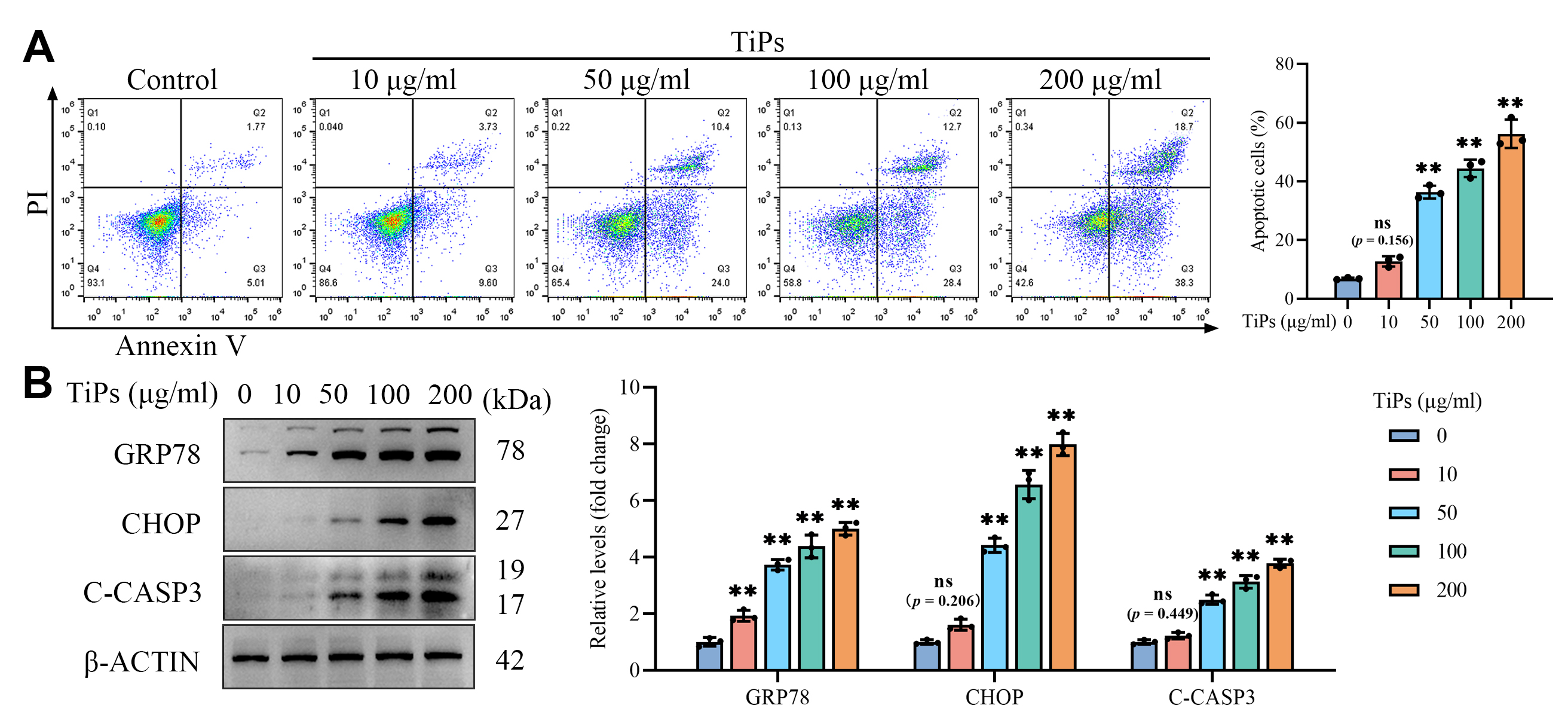


**Fig. S3. Dose-dependent induction of ER stress-mediated apoptosis in osteoblasts by TiPs exposure.** (**A**) Flow cytometry analysis of apoptosis levels in osteoblasts treated with various concentrations of TiPs (0, 10, 50, 100, and 200 μg/ml) for 24 h, using Annexin V/PI staining for detection. *n* = 3. (**B**) Western blot analysis of GRP78, CHOP, and C-CASP3 expression in osteoblasts treated as intended. *n* = 3. Data are presented as mean ± SD. One-way ANOVA with Tukey’s *post hoc* test. ^**^*P* < 0.01 *versus* the Control group. *ns*, not statistically significant *versus* the Control group.


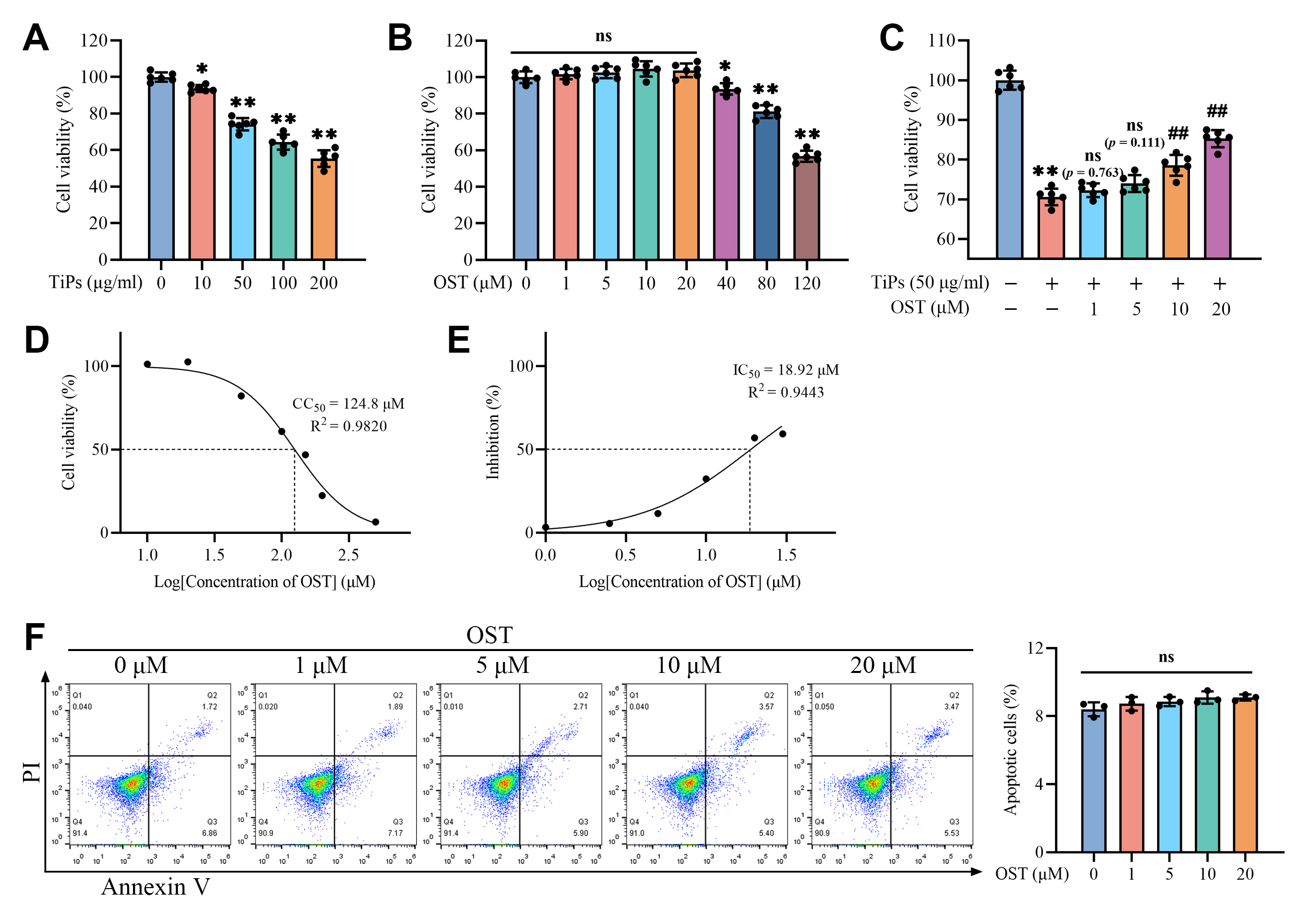


**Fig. S4. OST treatment rescued the reduction of cell viability in osteoblasts exposed to TiPs *in vitro*.** (**A**) Cell viability of osteoblasts treated with various concentrations of TiPs (0, 10, 50, 100, and 200 μg/ml) for 24 h, assessed using the CCK-8 assay. *n* = 6. (**B**) Cell viability of osteoblasts treated with various concentrations of OST (0, 1, 5, 10, 20, 40, 80, and 120 μM) for 24 h. *n* = 6. (**C**) Cell viability of osteoblasts exposed to TiPs (50 μg/ml) following treatment with various concentrations of OST (0, 1, 5, 10, and 20 μM) for 24 h. *n* = 6. (**D**) The CC50 of OST was determined by treating osteoblasts with various concentrations of OST (0, 10, 20, 100, 150, 200, 500 μM) for 24 h, assessed using the CCK-8 assay. *n* = 6. (**E**) The IC50 of OST for inhibiting TiPs cytotoxicity was determined by treating osteoblasts exposed to TiPs (50 μg/ml) with various concentrations of OST (0, 1, 2.5, 5, 10, 20, 30 μM) for 24 h, assessed using the CCK-8 assay. *n* = 6. (**F**) Flow cytometry analysis of apoptosis levels in osteoblasts treated with various concentrations of OST (0, 1, 5, 10, and 20 μM) for 24 h, using Annexin V/PI staining for detection. *n* = 3. Data are presented as mean ± SD. One-way ANOVA with Tukey’s *post hoc* test. ^*^*P* < 0.05 and ^**^*P* < 0.01 *versus* the Control group. ^##^*P* < 0.01 *versus* the TiPs group. *ns*, not statistically significant *versus* the Control or TiPs group.


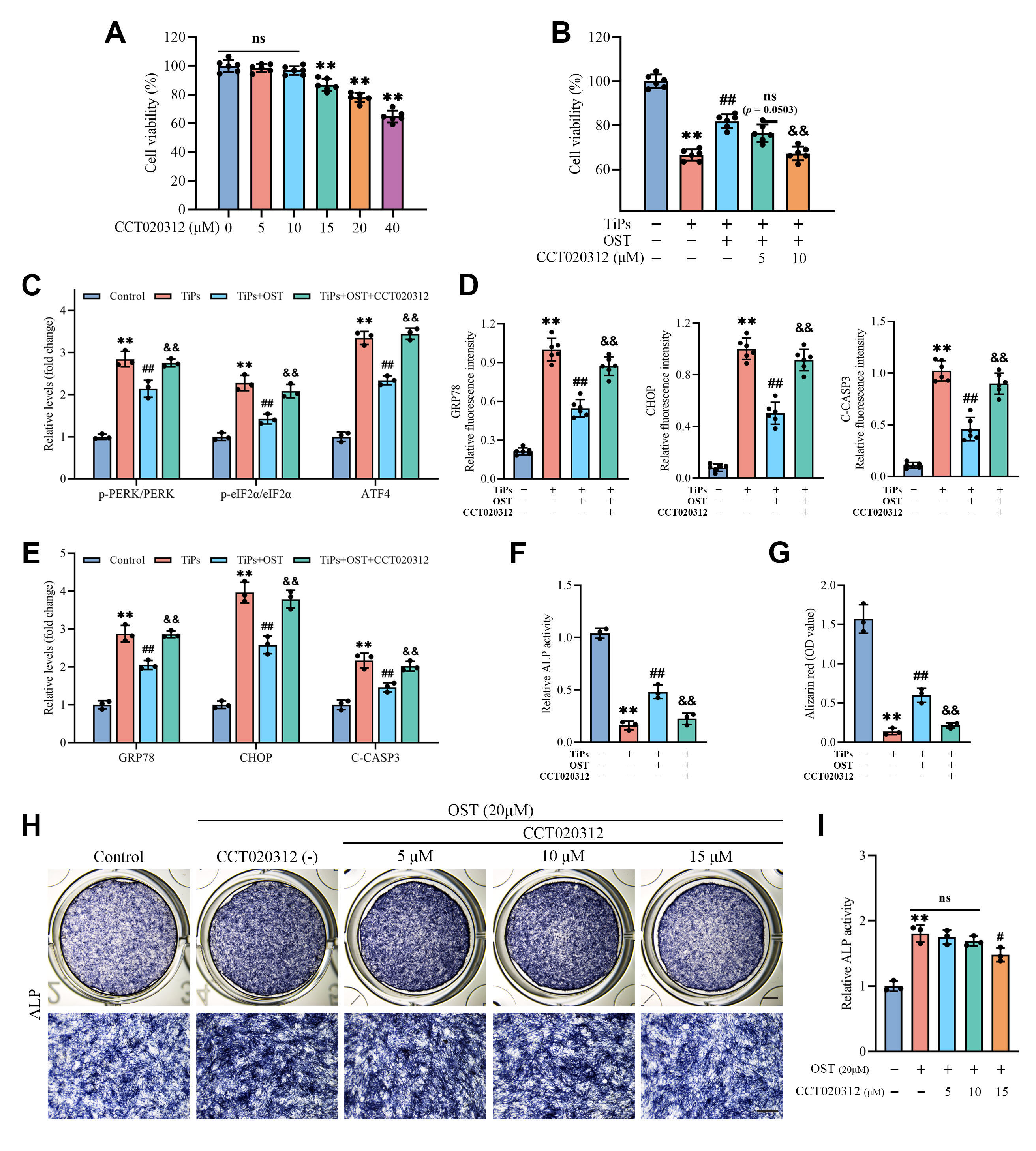


**Fig. S5. CCT020312 abolished the protective effects of OST on stress osteoblasts upon TiPs exposure *in vitro*.** (**A**) Cell viability of osteoblasts treated with various concentrations of CCT020312 (0, 5, 10, 15, 20, and 40 μg/ml) for 24 h, assessed using the CCK-8 assay. *n* = 6. (**B**) Cell viability of osteoblasts exposed to TiPs (50 μg/ml) following treatment with OST (20 μM) or OST combined with CCT020312 (5 or 10 μM) for 24 h. *n* = 6. (**C**) Western blot analysis of the protein levels of PERK, p-PERK, eIF2α, p-eIF2α, and ATF4 in osteoblasts exposed TiPs (50 μg/ml) following treatment with OST (20 μM) or OST combined with CCT020312 (10 μM) for 24 h. *n* = 3. (**D**) Quantitative analysis of GRP78, CHOP, and C-CASP3 immunofluorescence staining results in osteoblasts treated as intended. *n* = 6. (**E**) Western blot analysis of the protein levels of GRP78, CHOP, and C-CASP3 in osteoblasts treated as intended. *n* = 3. (**F**) Quantitative analysis of the results of ALP staining. *n* = 3. (**G**) Quantitative analysis of the results of ARS staining. *n* = 3. (**H**) Representative images of ALP staining of osteoblasts treated with OST (20 μM) or OST combined with CCT020312 (5, 10, or 15 μM) following 7 days of osteogenic induction. Scale bar, 2 mm (upper), 500 μm (lower). (**I**) Quantitative analysis of relative ALP activity. *n* = 3. Data are presented as mean ± SD. One-way ANOVA with Tukey’s *post hoc* test. ^**^*P* < 0.01 *versus* the Control group. ^#^*P* < 0.05 *versus* the OST alone group. ^##^*P* < 0.01 *versus* the TiPs group. ^&&^*P* < 0.01 *versus* the TiPs + OST group. *ns*, not statistically significant *versus* the OST alone group.

**Uncropped Western blots and Agarose gels.**


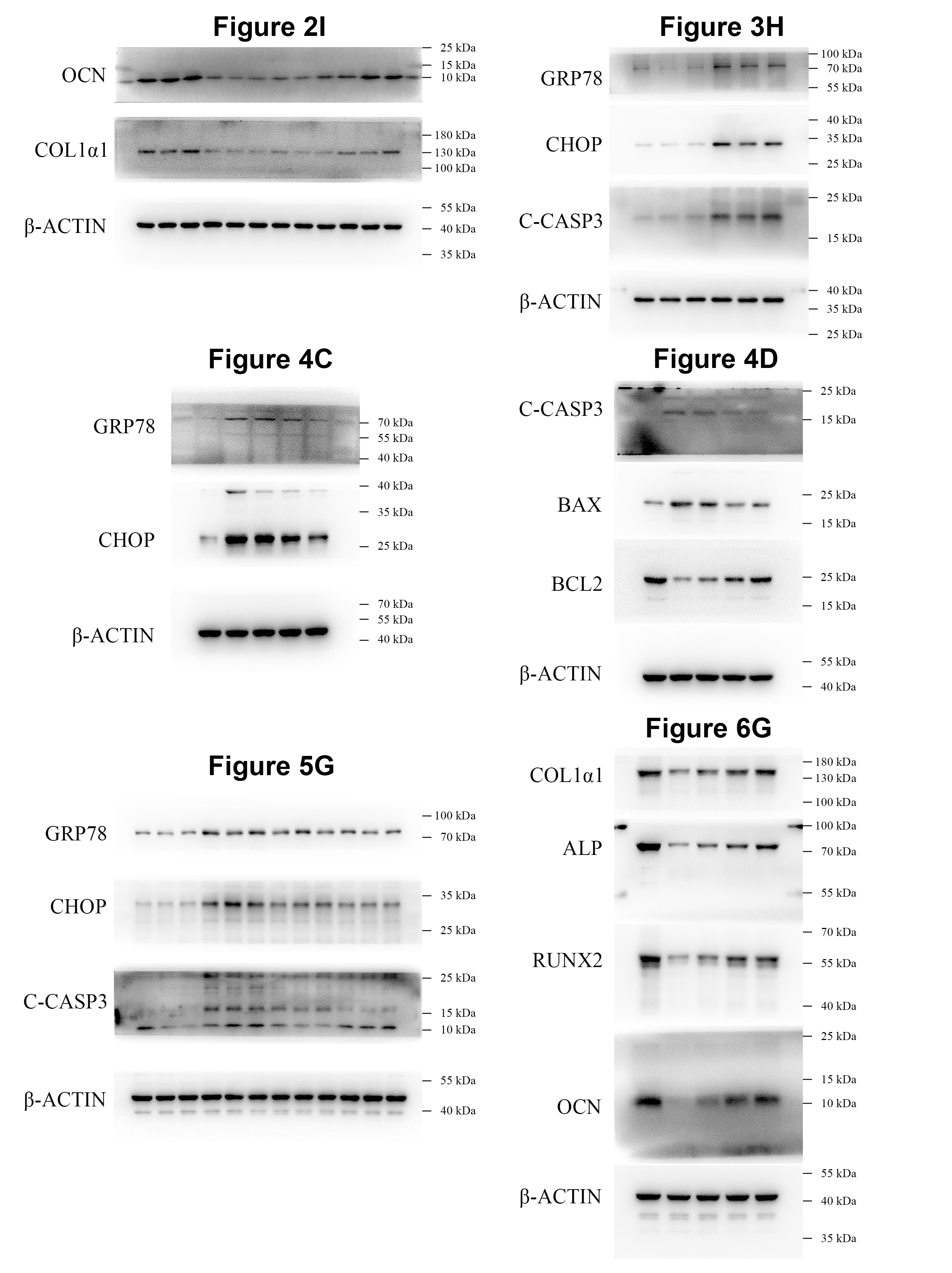


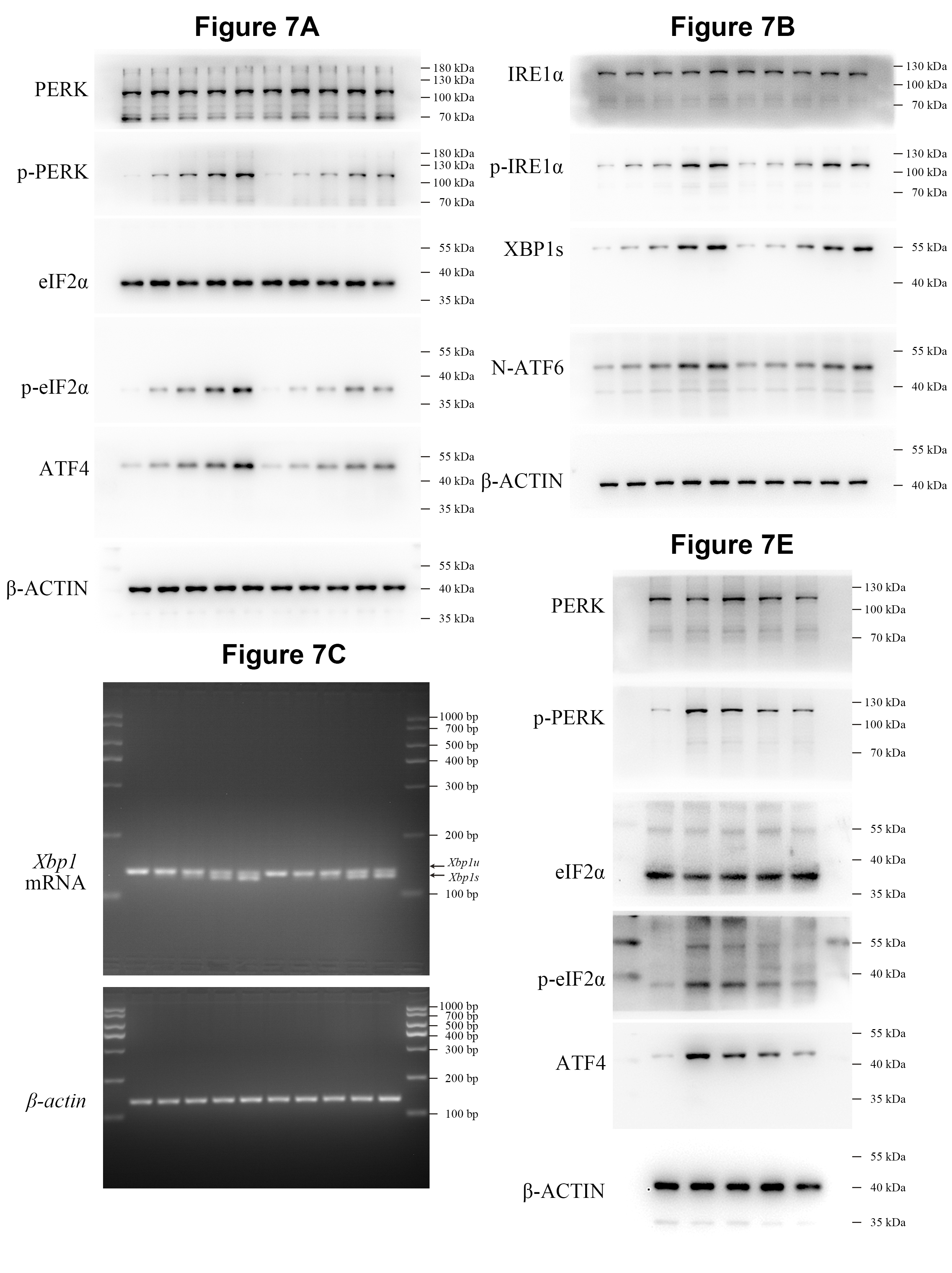


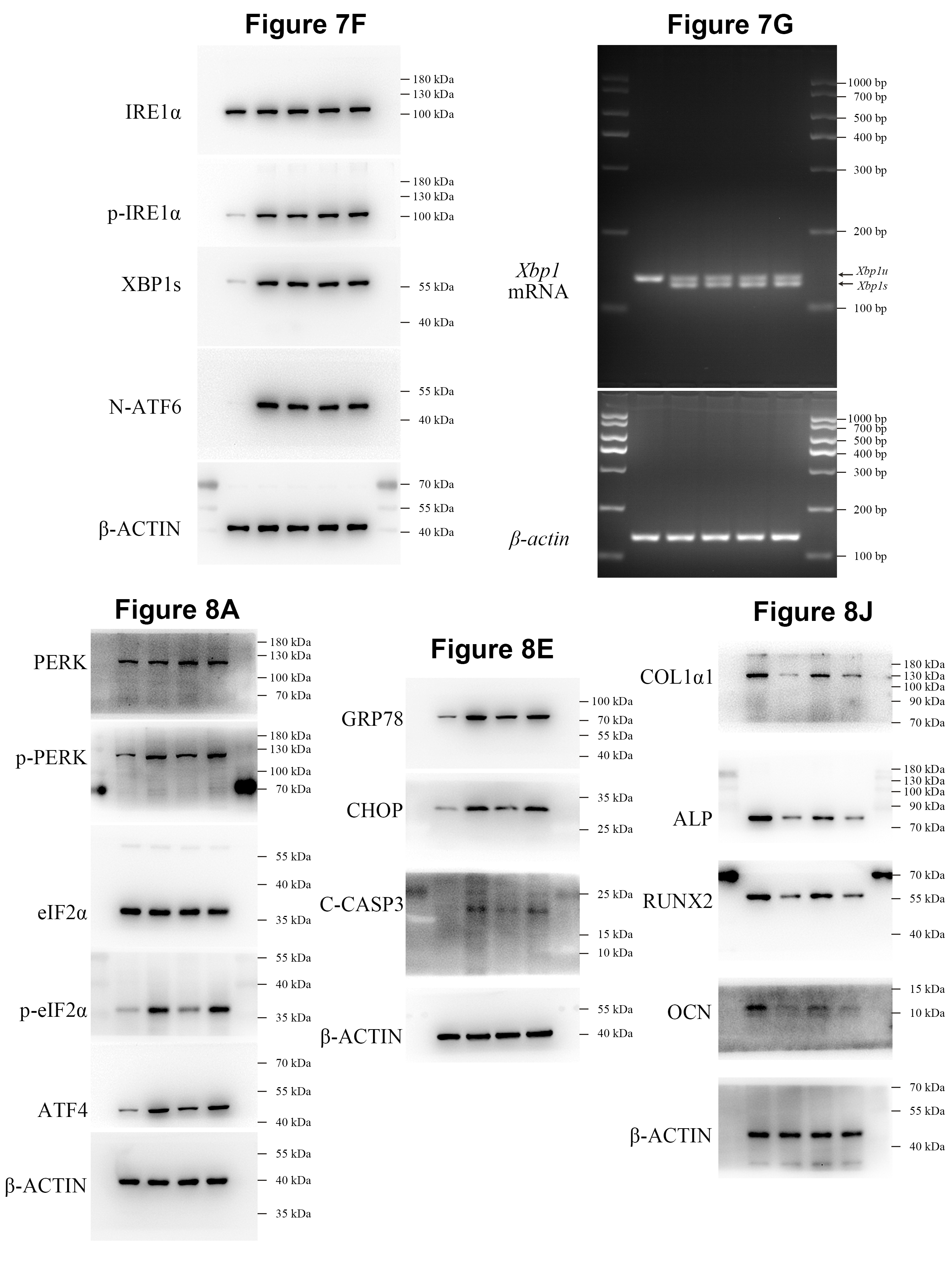


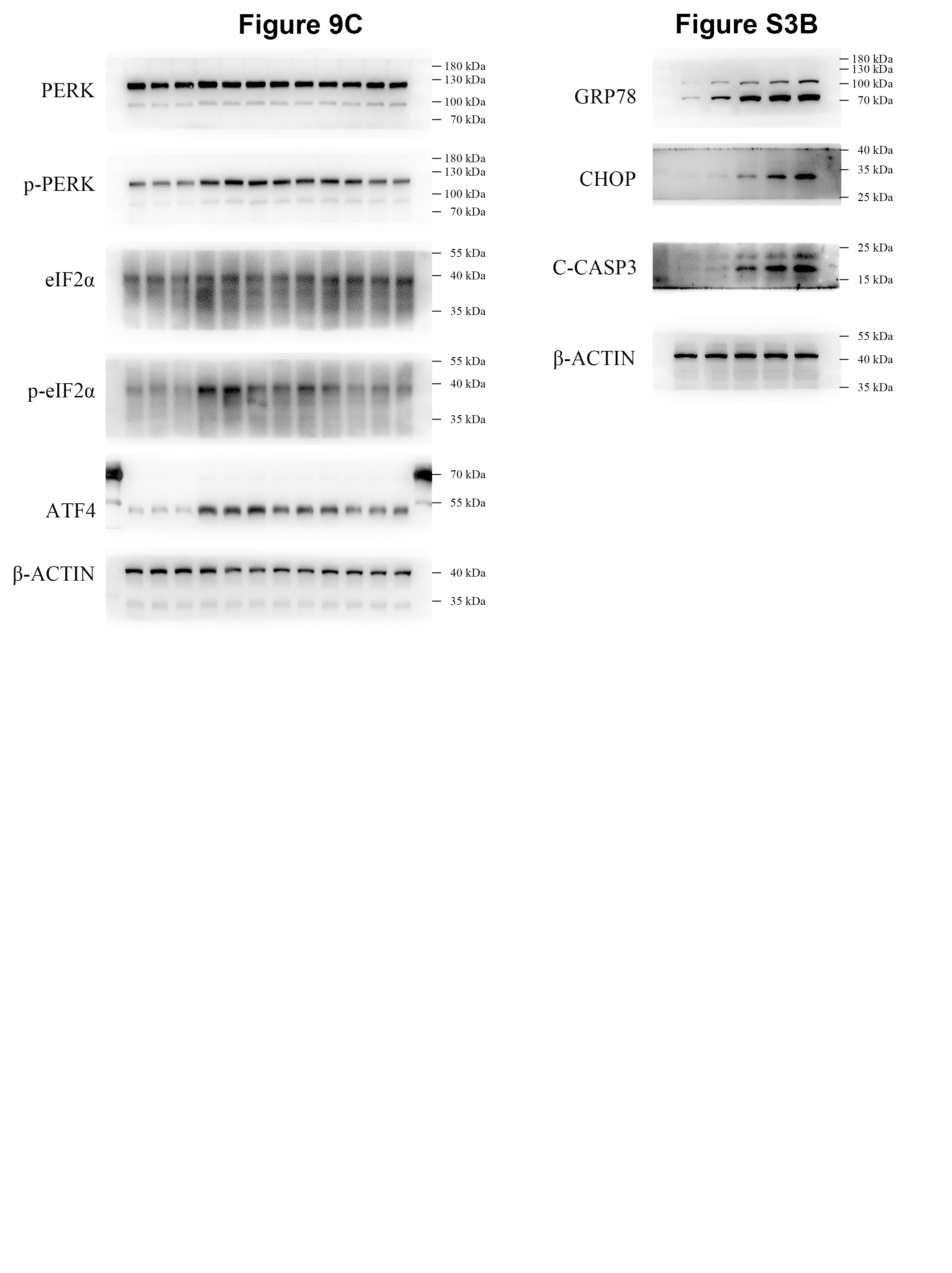

Supplement: Supplementary file 1 — Supplementary Material 1 [file 10020_2024_1034_MOESM1_ESM.docx]
